# Supplementary figures and images for: Preprocessing choices affect RNA velocity results for droplet scRNA-seq data
Source: PLoS Comput Biol. 2021 Jan 11;17(1):e1008585. doi: 10.1371/journal.pcbi.1008585 (PMC7822509; doi:10.1371/journal.pcbi.1008585)

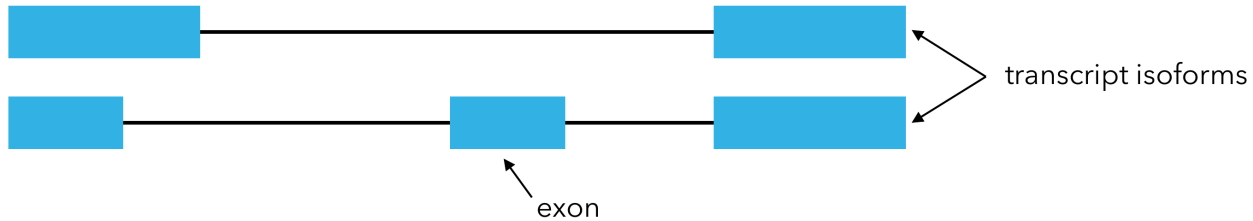

## Collapse

flanking  
sequence

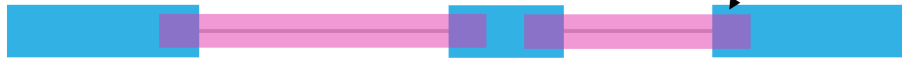

## Separate

intron

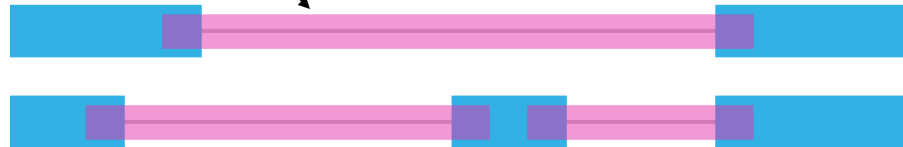

Supplement: S1 Fig — With the ‘collapse’ approach, the annotated isoforms of a gene are first collapsed before the introns are defined as any non-exonic region of the gene locus. With the ‘separate’ approach, introns are separately defined for each isoform. After extracting the intronic regions, a flanking region is added to each side of the introns to accommodate reads overlapping exon/intron boundaries. (PDF) [file pcbi.1008585.s001.pdf]

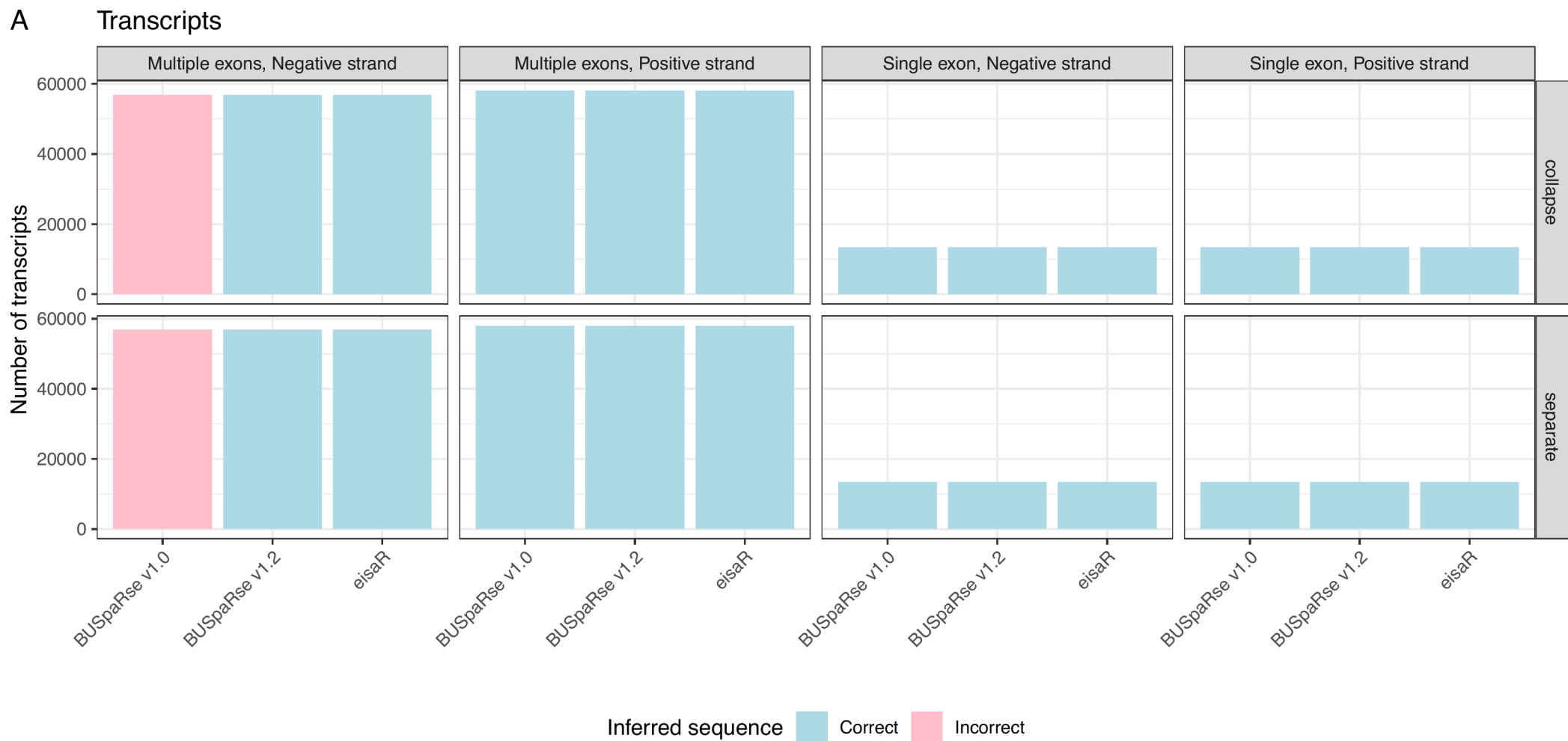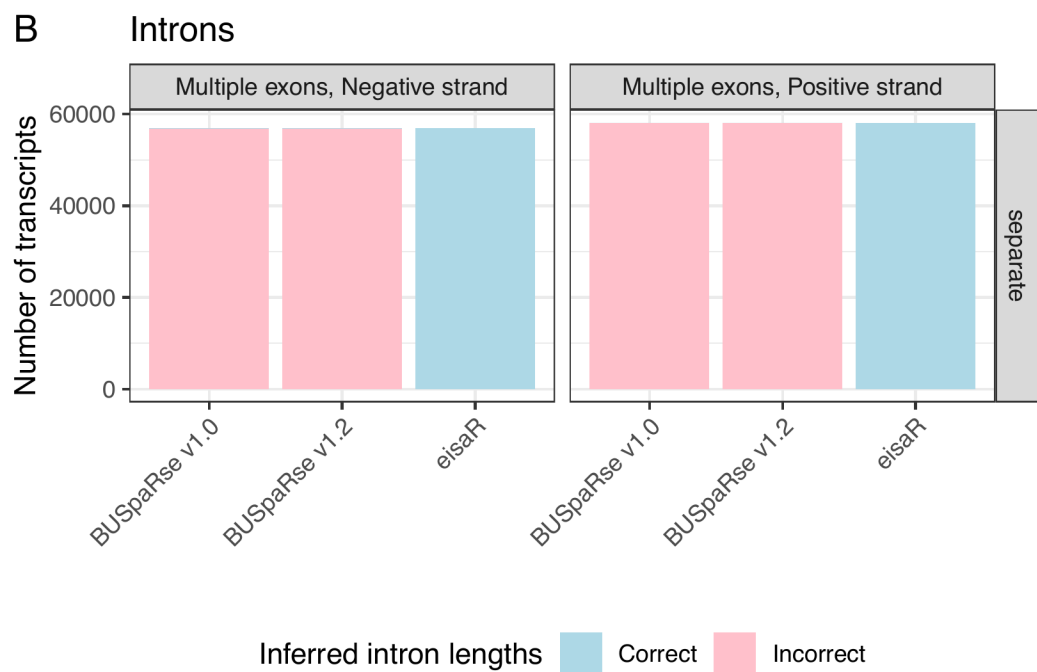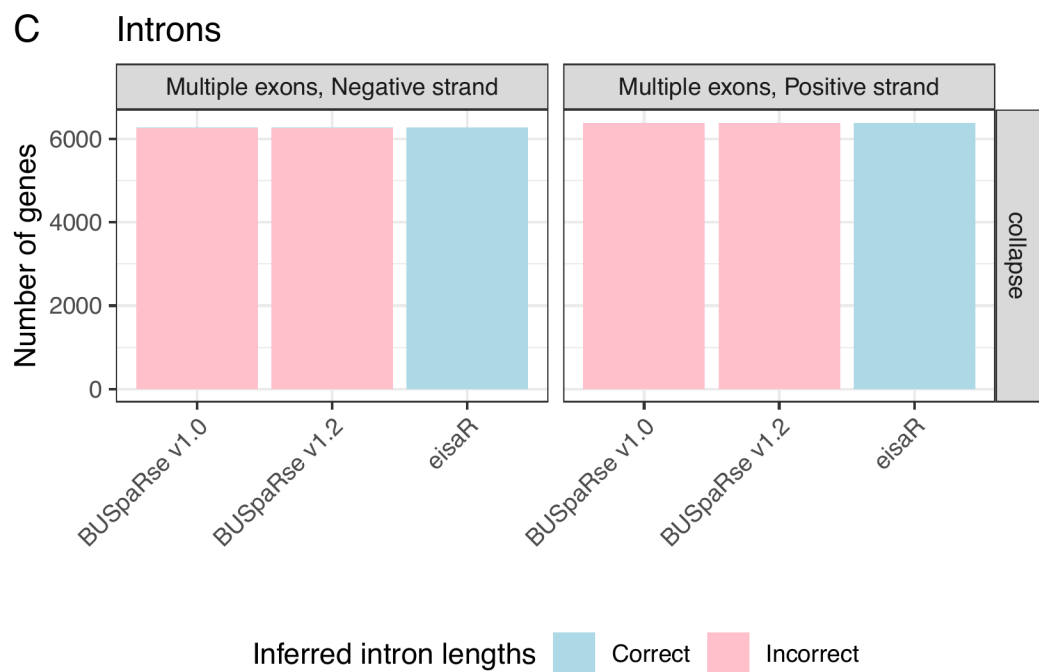

Supplement: S2 Fig — A. The number of transcripts extracted by BUSpaRse and eisaR that have the correct or incorrect sequence, respectively, as determined by comparing to the transcriptome fasta file downloaded from Gencode. Transcripts are stratified by whether or not they are multi-exonic, and by the strand. B. The number of transcripts for which the lengths of the introns inferred by BUSpaRse and eisaR are correct or incorrect, respectively, as determined by comparing to the introns extracted by the intronsByTranscript function from the GenomicFeatures Bioconductor package (‘separate’ intron definition). C. The number of single-transcript genes for which the lengths of the introns inferred by BUSpaRse and eisaR are correct or incorrect, respectively, as determined by comparing to the introns extracted by the intronsByTranscript function from the GenomicFeatures Bioconductor package (‘collapse’ intron definition). (PDF) [file pcbi.1008585.s002.pdf]

OldBrain, total count, stratified by overall uniqueness (collapse)

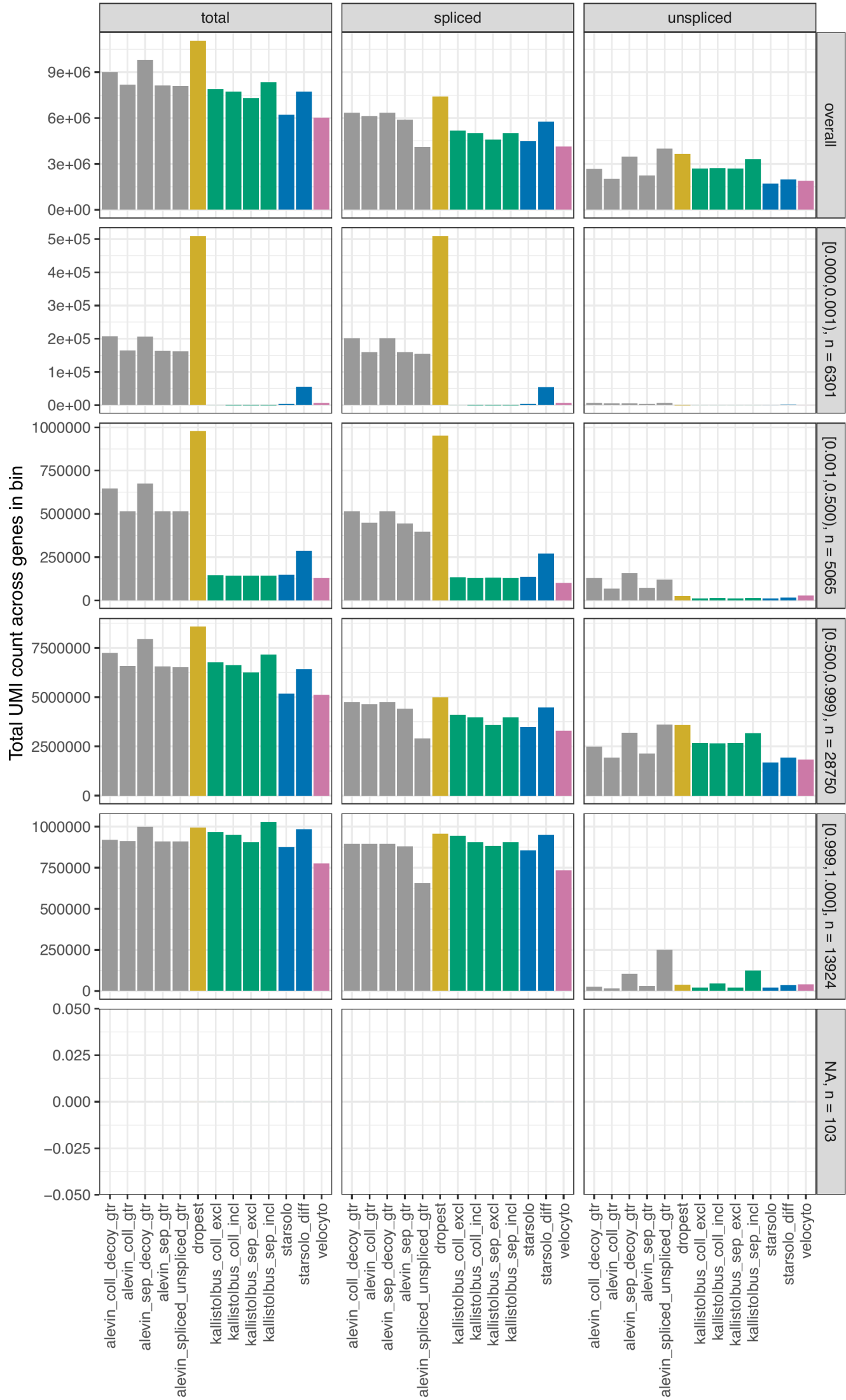

Supplement: S4 Fig — The bars correspond to the total UMI count, and the split of these into counts for exonic and intronic targets, for each quantification method in the OldBrain data set. In addition to the overall count (top row), the figure shows the total count after stratifying genes by the overall fraction of unique k-mers (using the ‘collapse’ annotation), indicated in the vertical panel headers together with the number of genes in the category. The genes for which no uniqueness information could be calculated (the ‘NA’ category) are those for which all transcripts are shorter than the chosen k-mer length (which was set to the read length minus one; here 56nt). (PDF) [file pcbi.1008585.s004.pdf]

Pancreas, differences in total gene count across cells

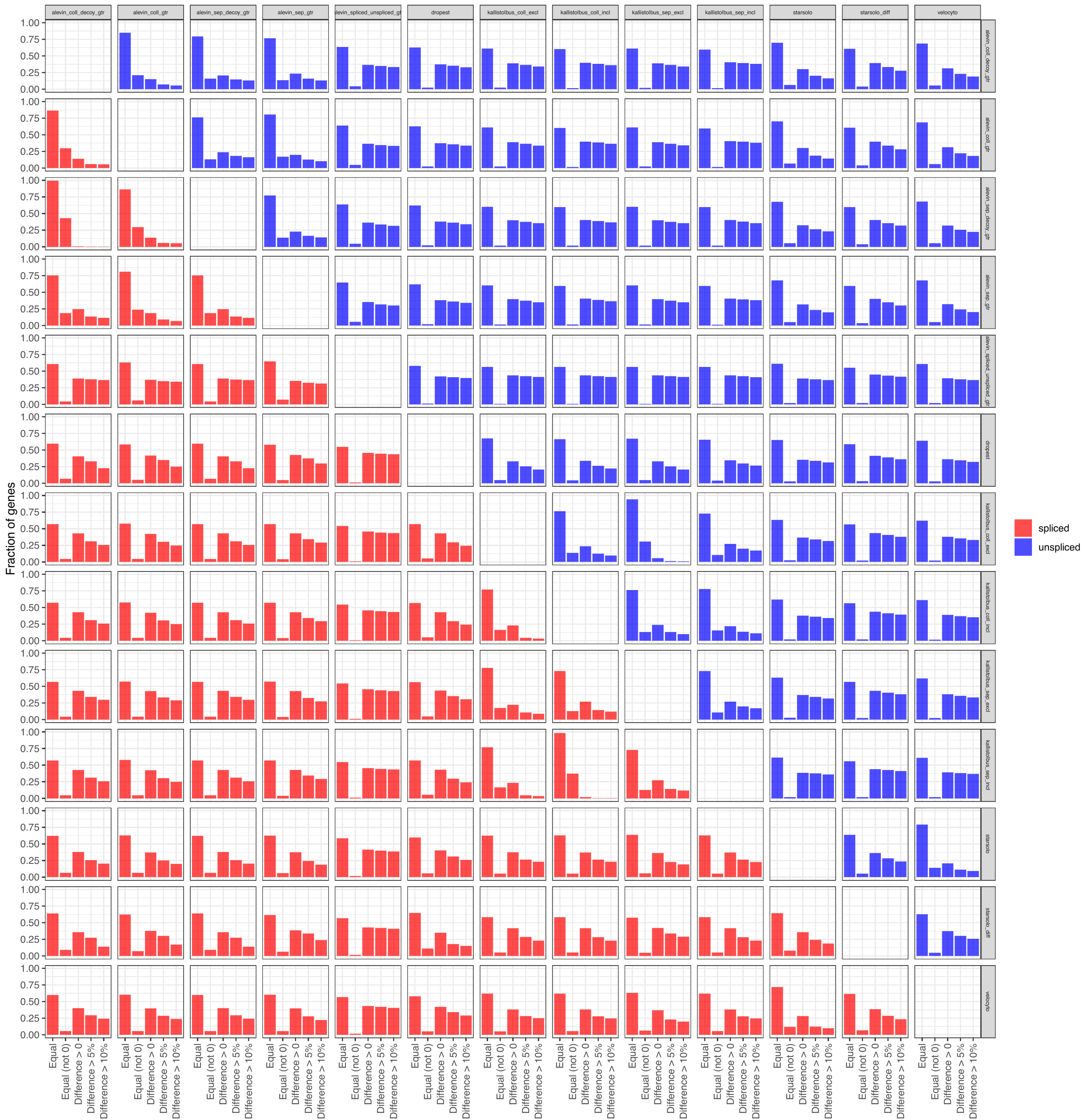

Supplement: S5 Fig — For each pair of methods, the figure shows the fraction of genes that obtain the same count with the two methods (both overall and after excluding the genes assigned a count of 0 with both methods) as well as the fraction of genes where the difference between the assigned counts is non-zero, greater than 5% (of the average count assigned by the two methods) or greater than 10%. (PDF) [file pcbi.1008585.s005.pdf]

Pancreas

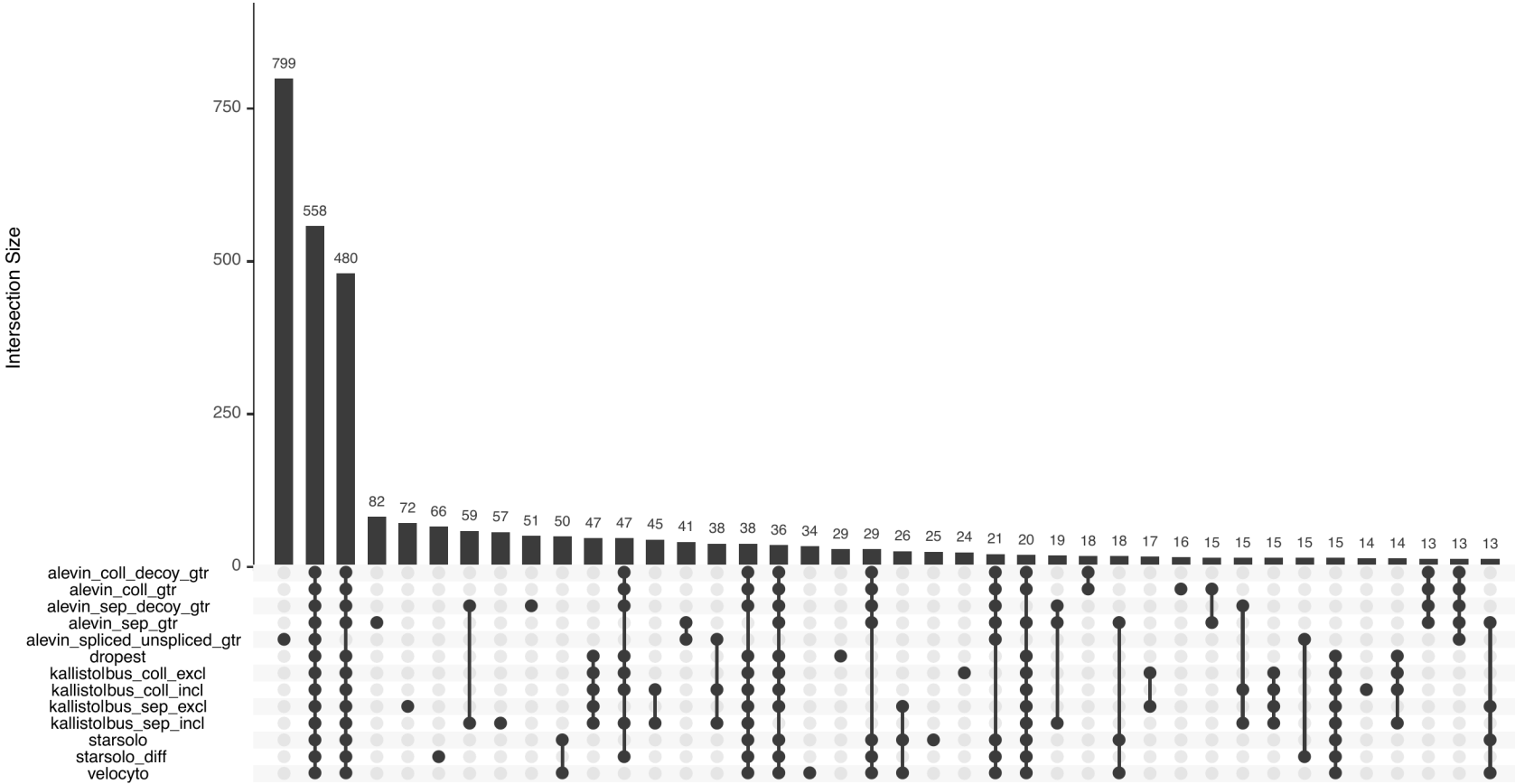

Spermatogenesis

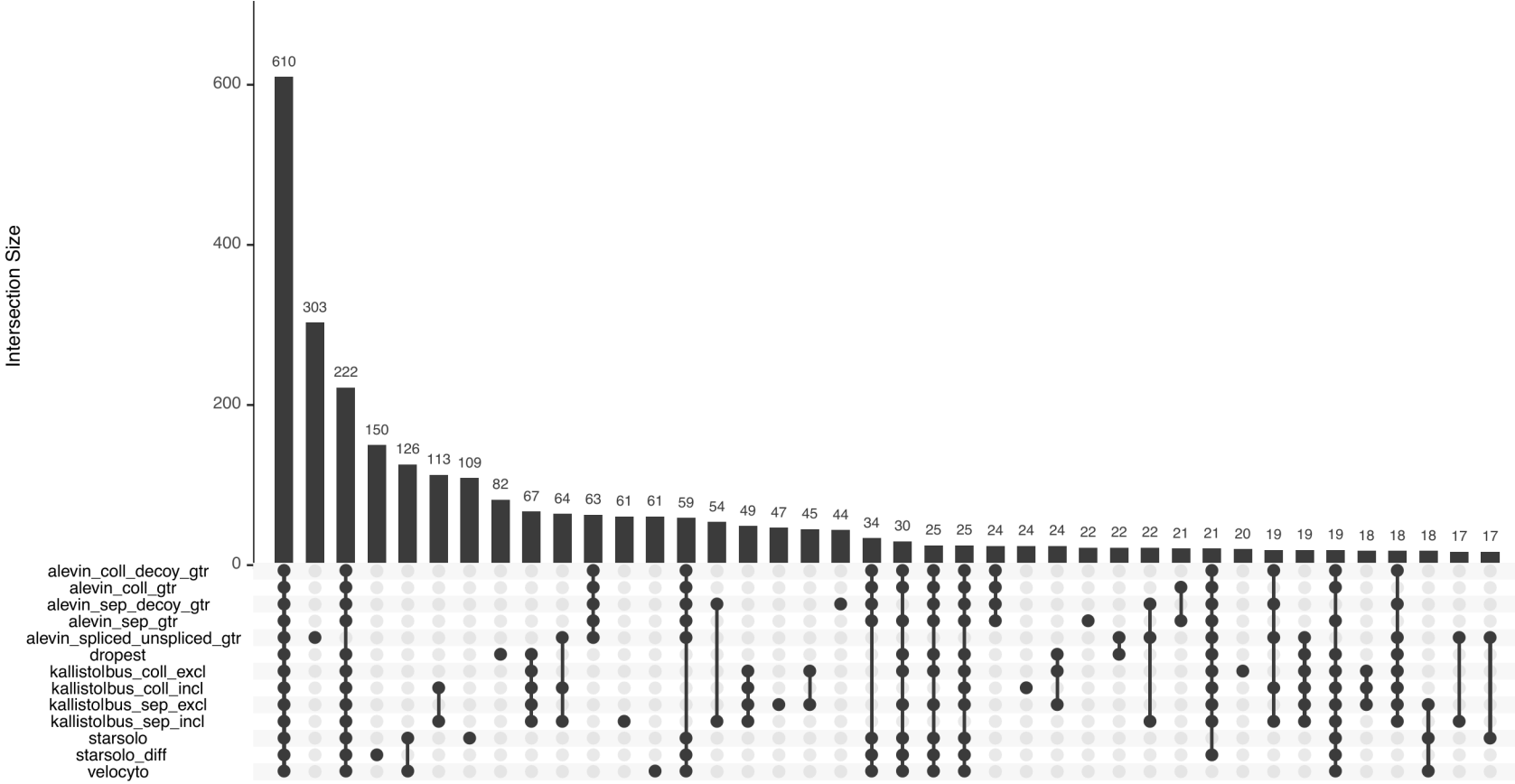

Dentate gyrus

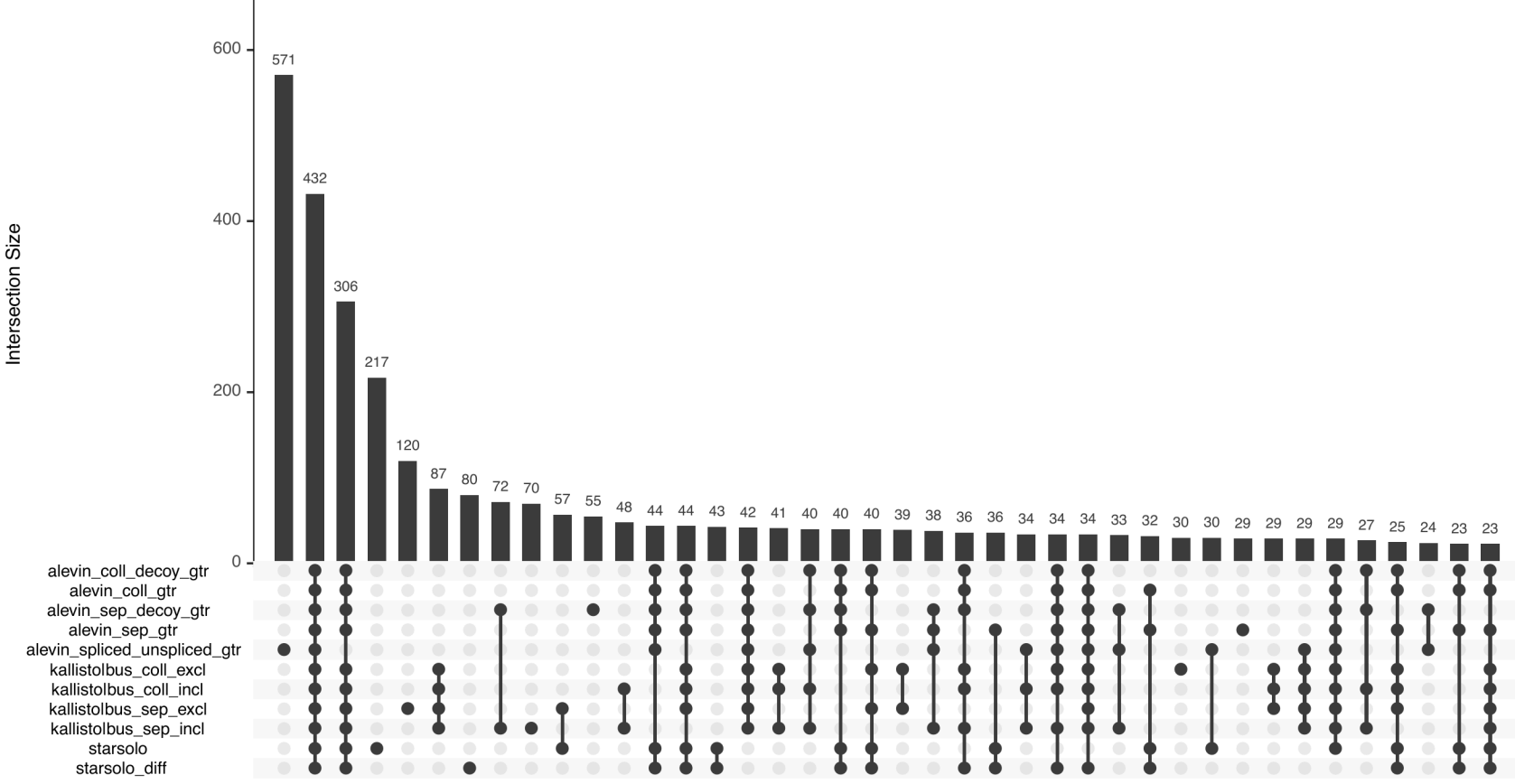

Supplement: S6 Fig — Each column corresponds to the number of genes shared by a particular set of methods (indicated by black dots). (PDF) [file pcbi.1008585.s006.pdf]

ENSMUSG00000032324 (Tspan3)

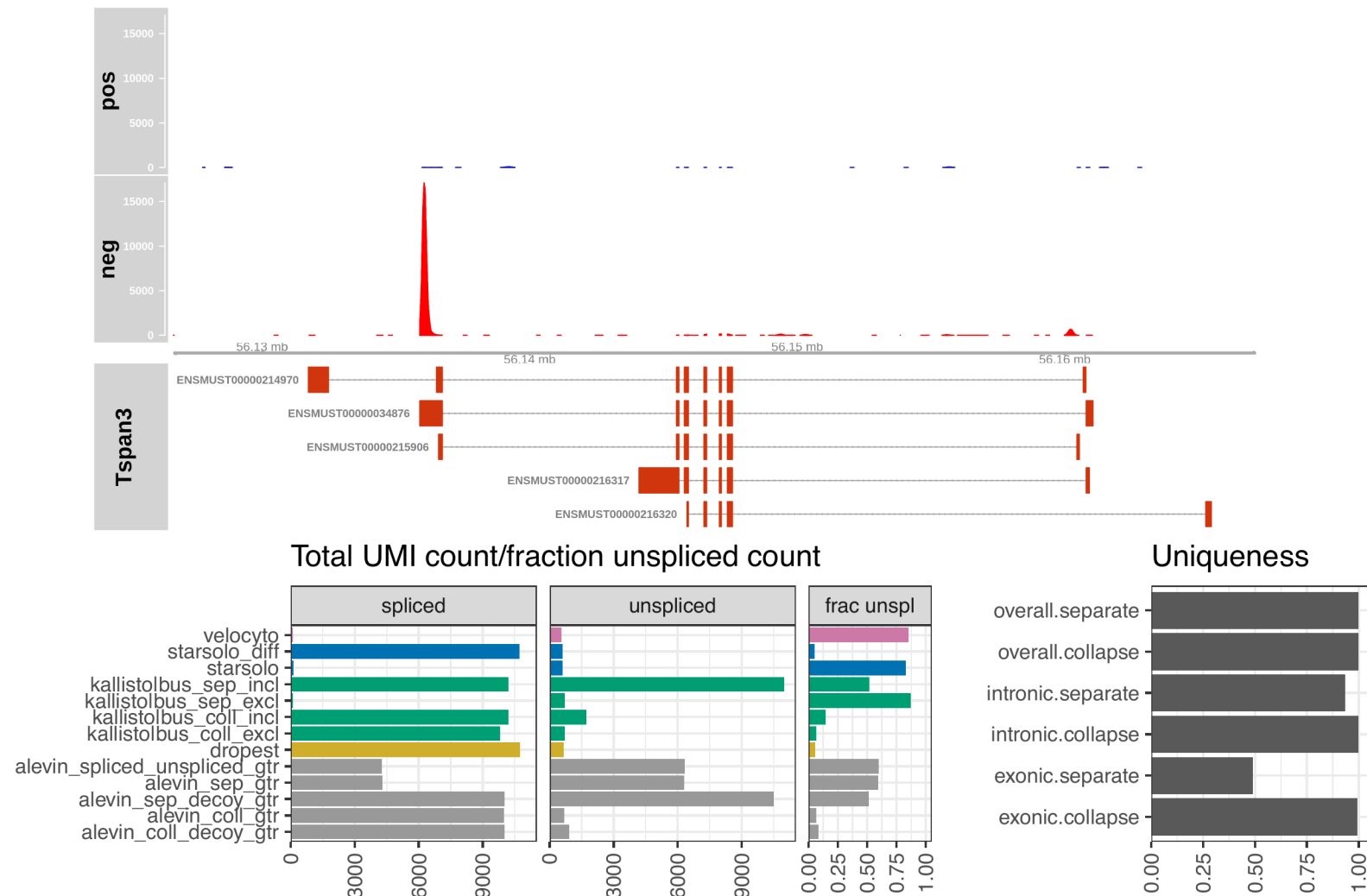

ENSMUSG00000021427 (Ssr1)

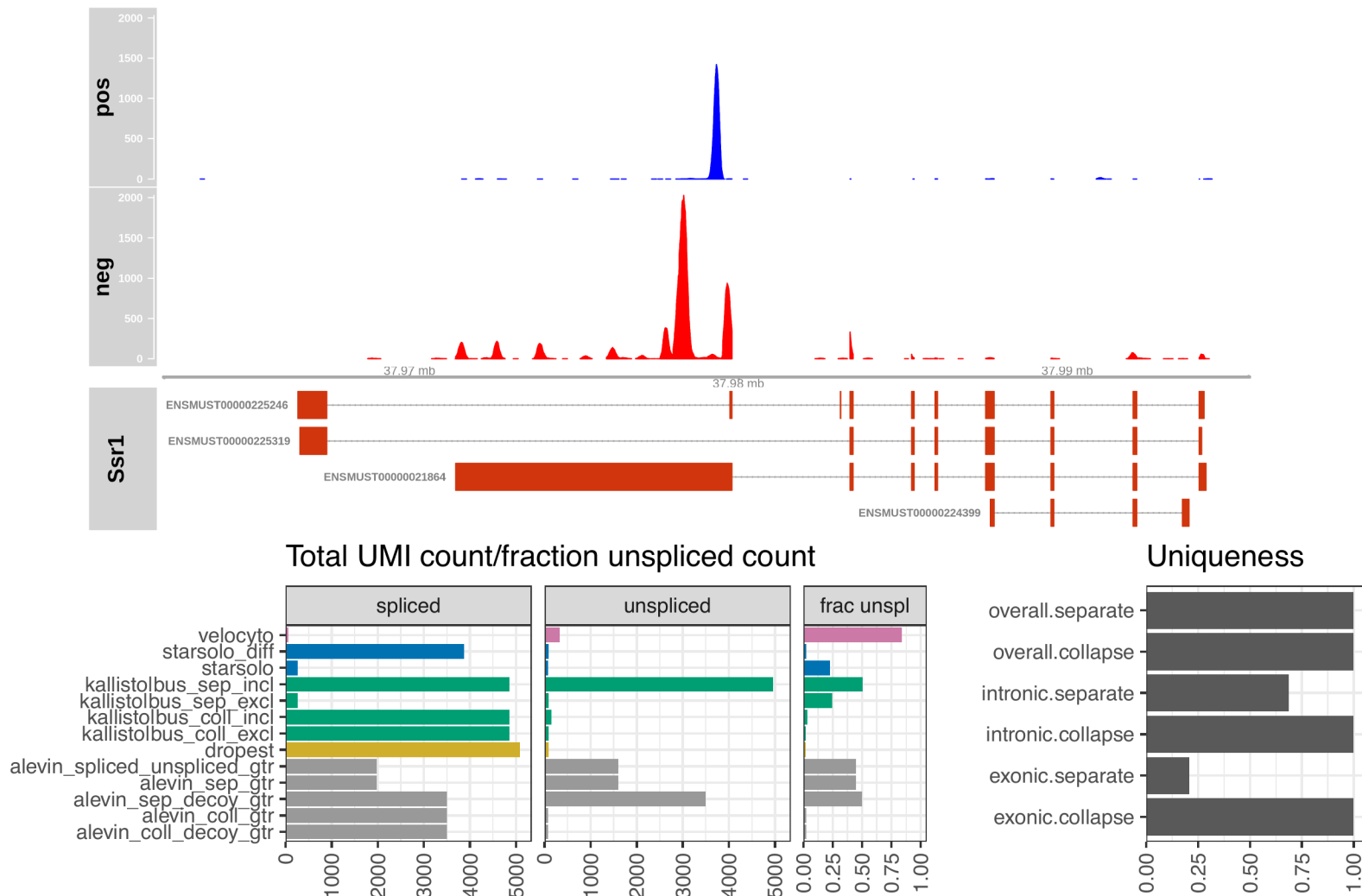

ENSMUSG00000039740 (Alg2)

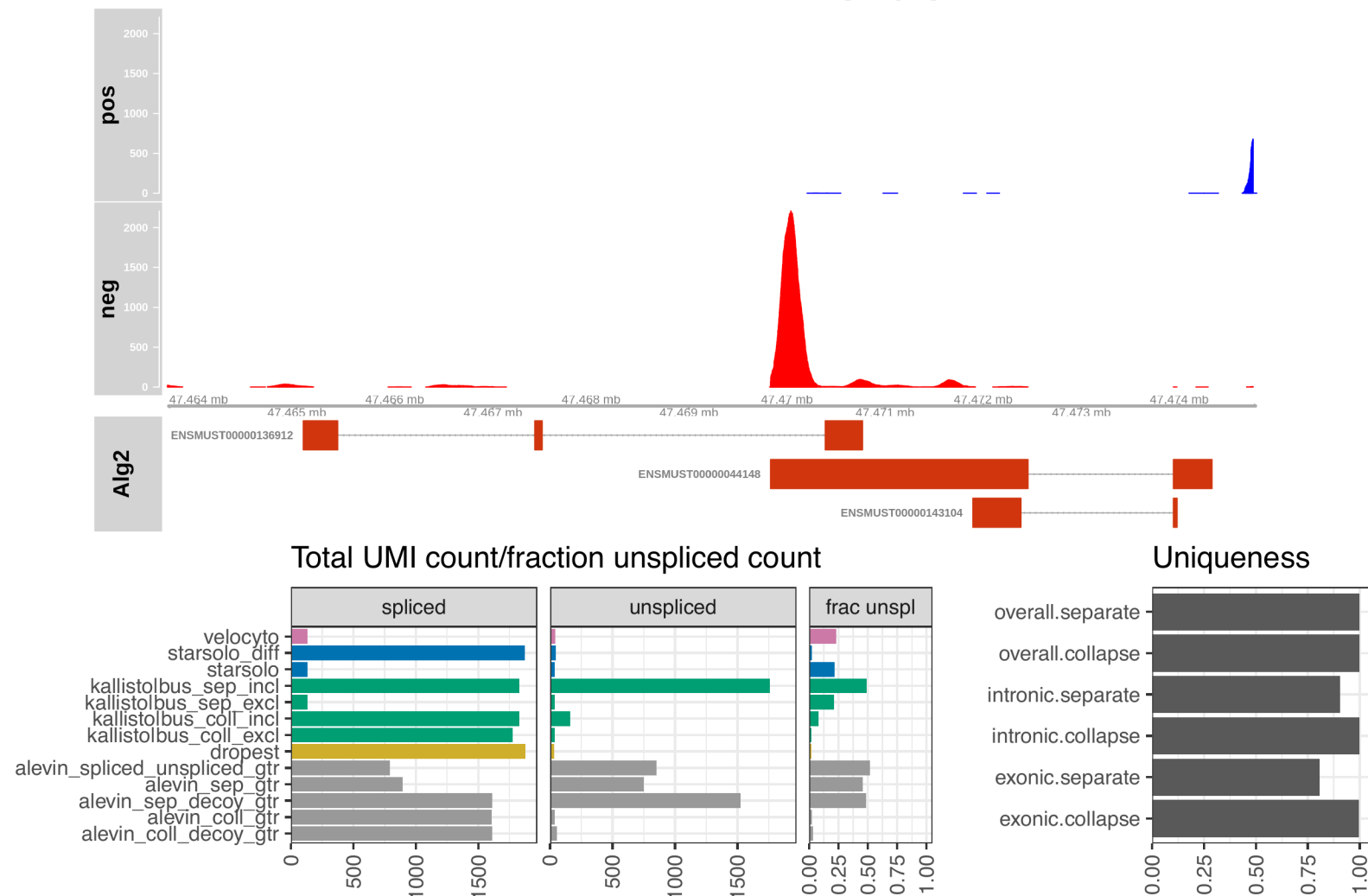

ENSMUSG00000053046 (Brsk2)

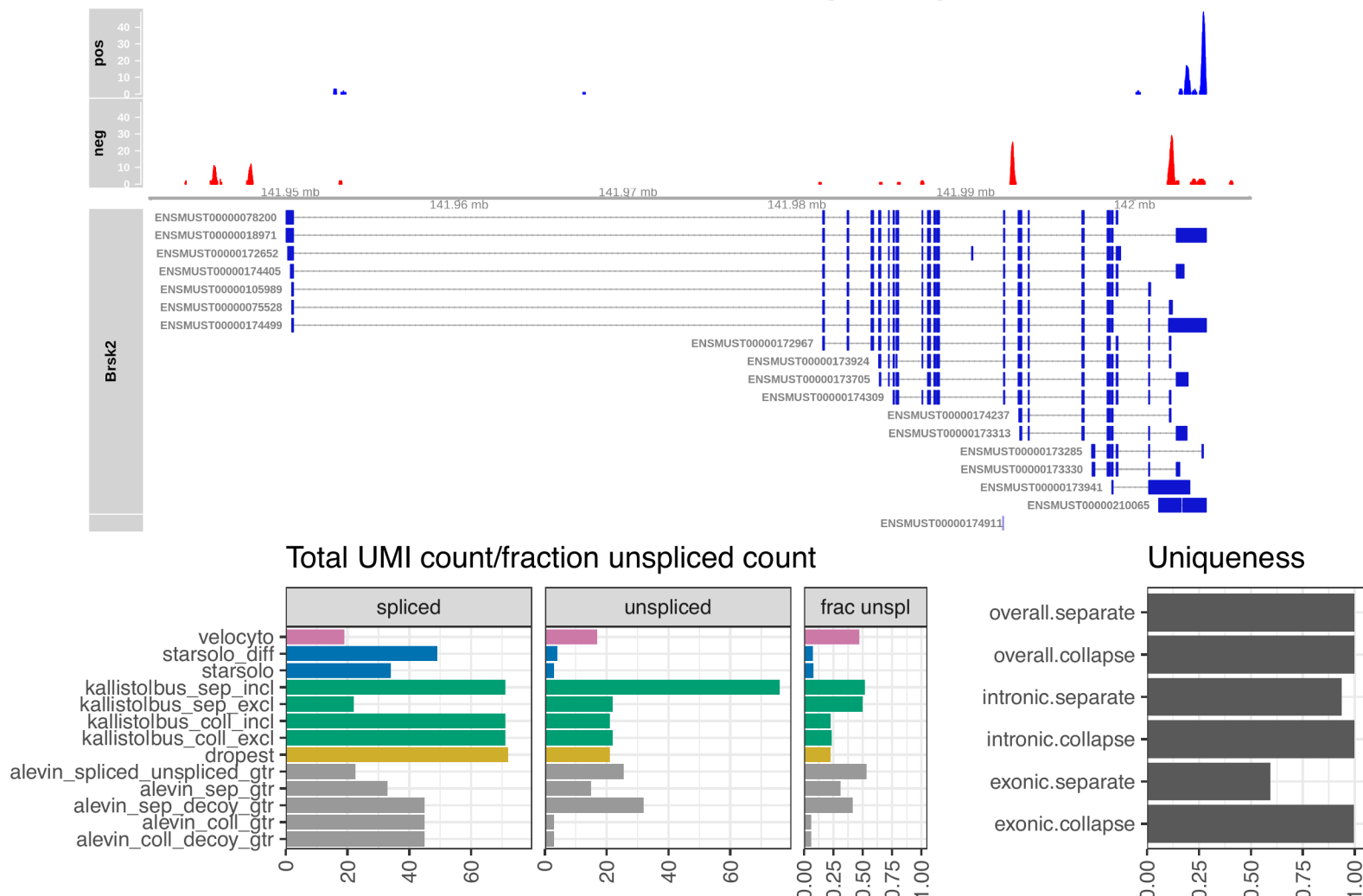

ENSMUSG00000086390 (1810019D21Rik)

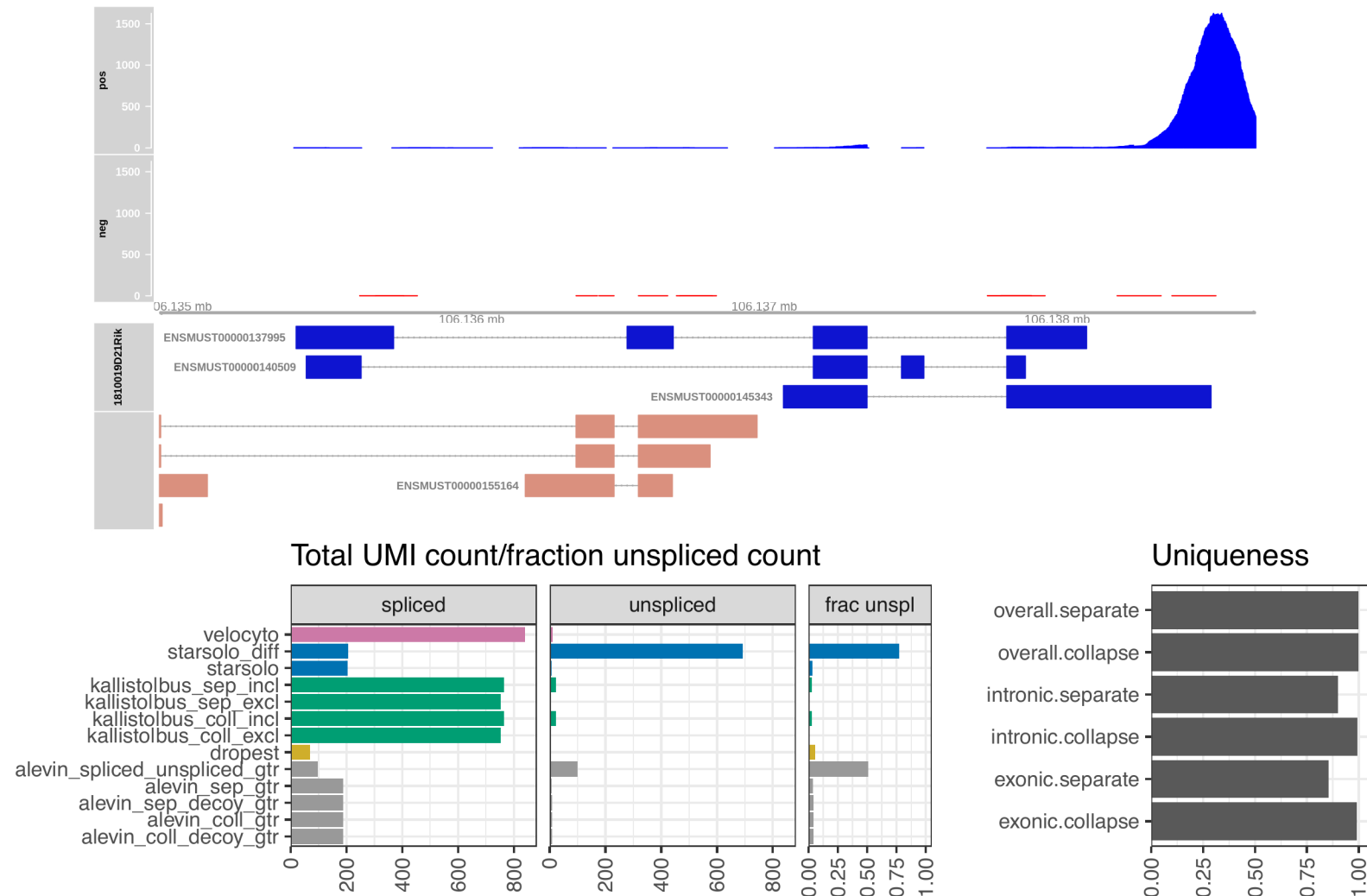

ENSMUSG00000052727 (Map1b)

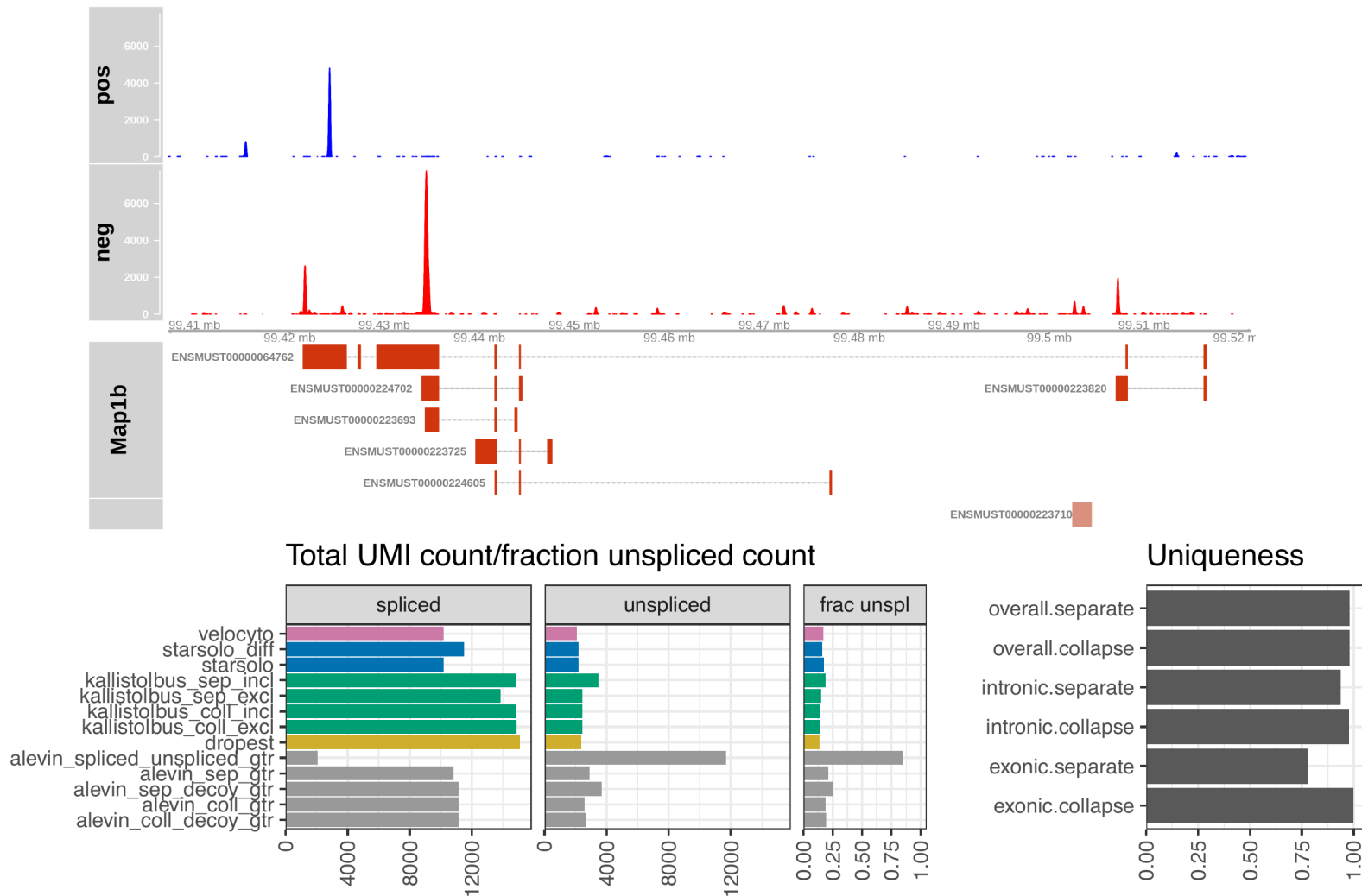

Supplement: S7 Fig — The coverage tracks show the number of reads overlapping each base position, on the positive (blue) or negative (red) strand. The isoforms of the main gene in focus of each panel are similarly shown in blue (positive strand) or red (negative strand). Any overlapping features from other genes are shown in the bottom annotation track, colored in muted blue or red, depending on the annotated strand. The bottom panels show the total exonic and intronic UMI count assigned to the displayed gene by the different quantification methods, as well as the fraction of unspliced counts, and the fraction of unique k-mers in the gene overall, as well as in the exons and introns. (PDF) [file pcbi.1008585.s007.pdf]

ENSMUSG00000022617 (Chkb)

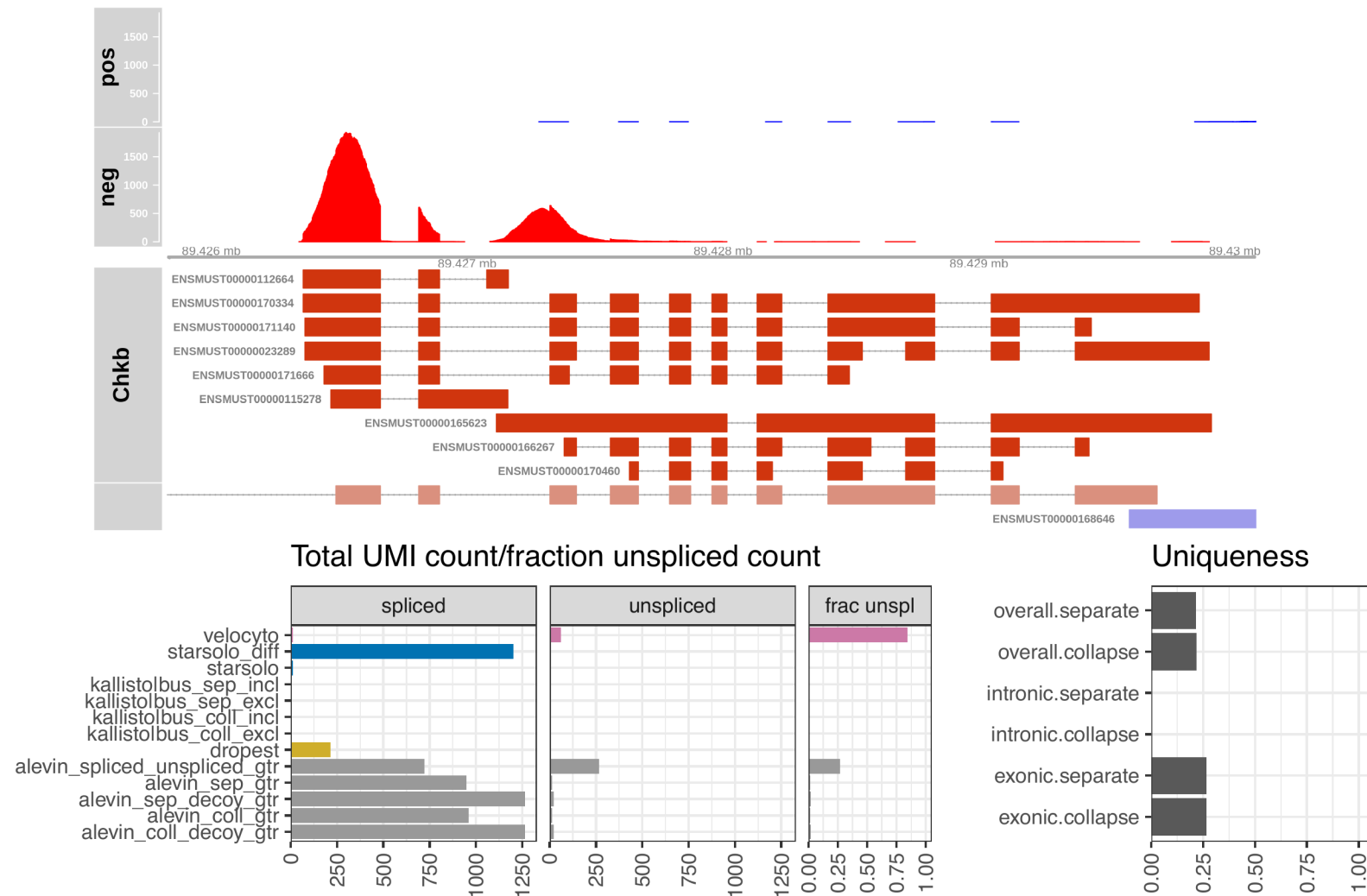

ENSMUSG00000095538 (Gm21983)

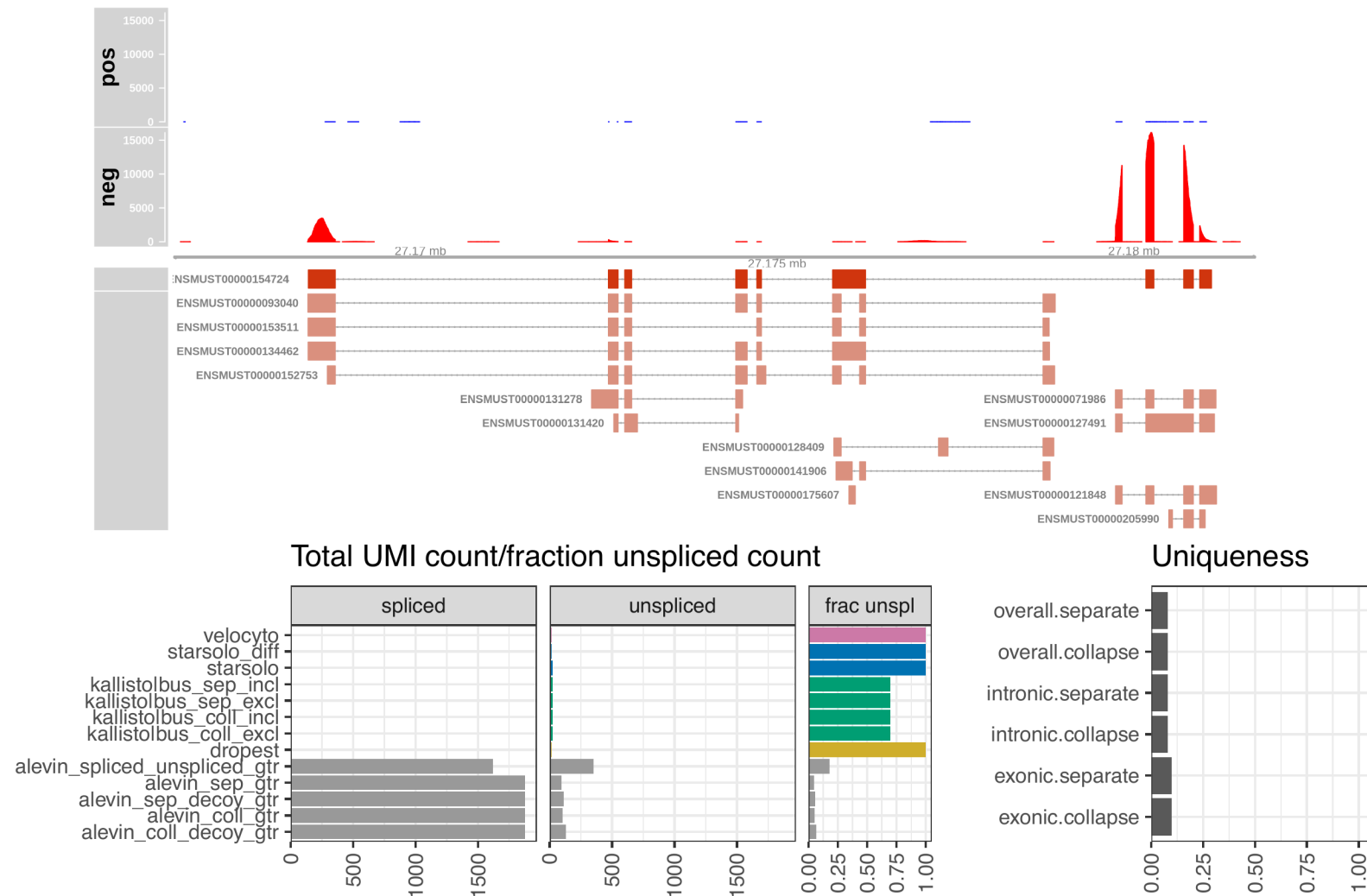

ENSMUSG00000010067 (Rassf1)

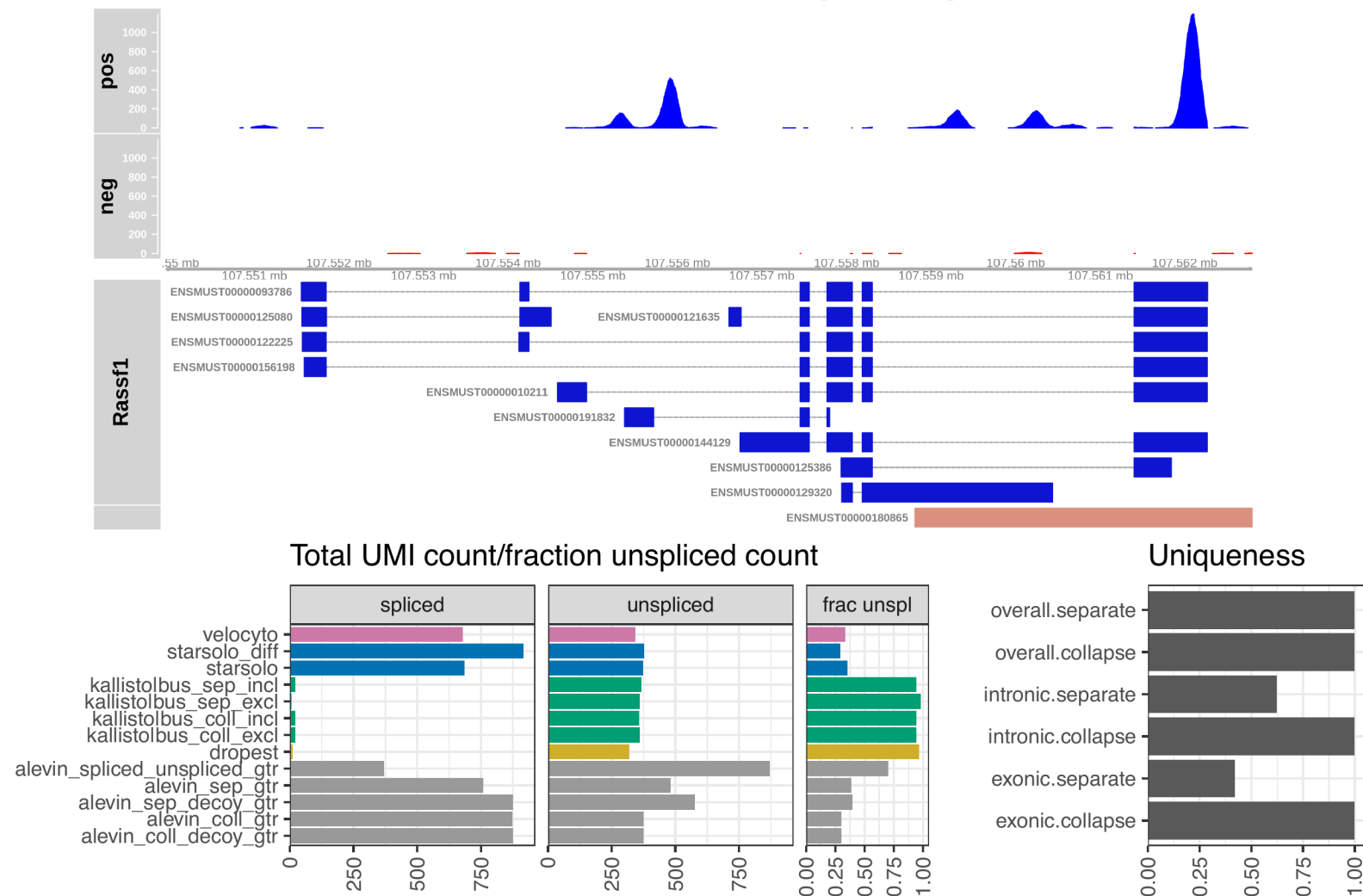

ENSMUSG00000020362 (Cnot6)

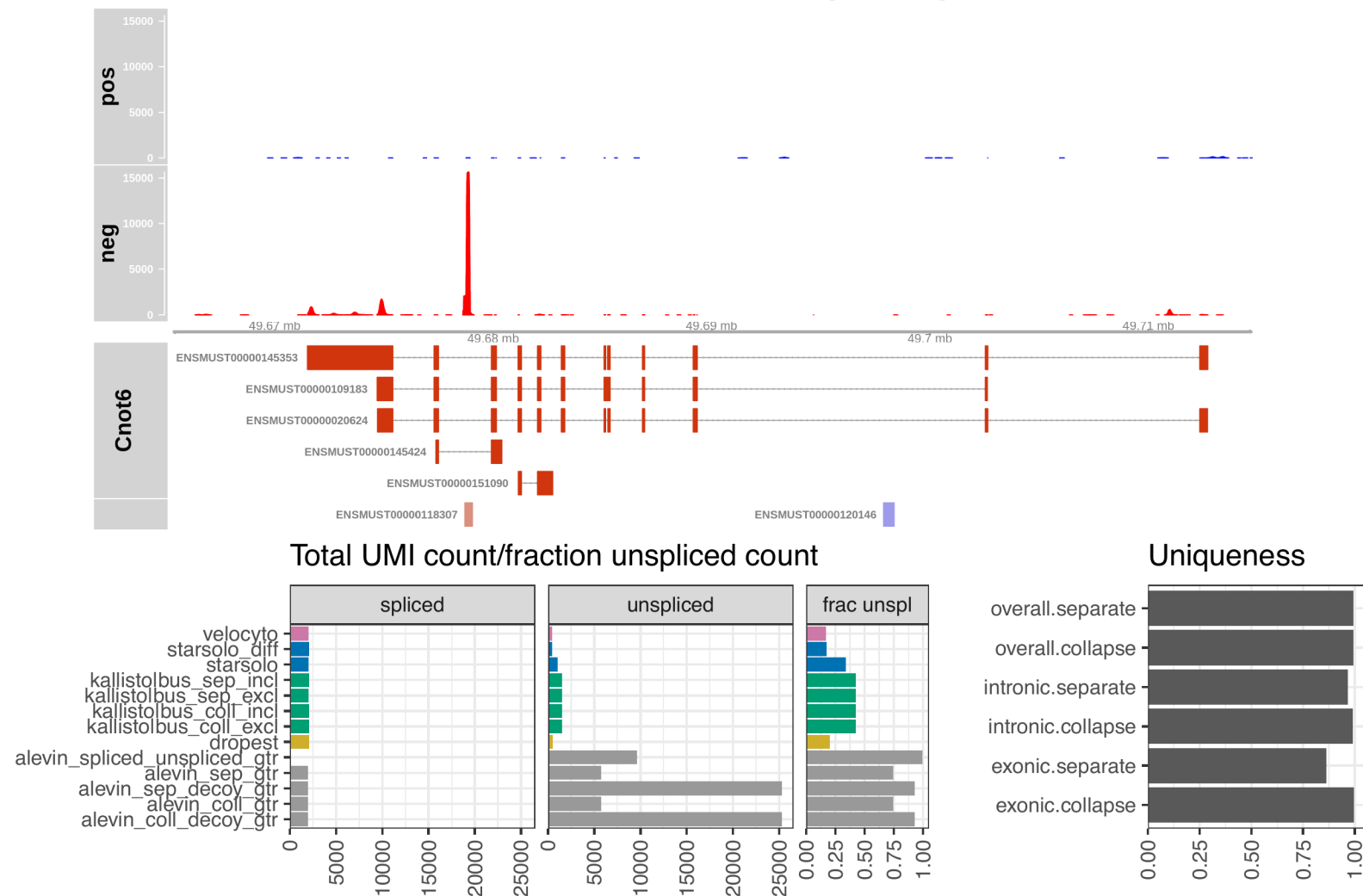

ENSMUSG00000083061 (Gm12191)

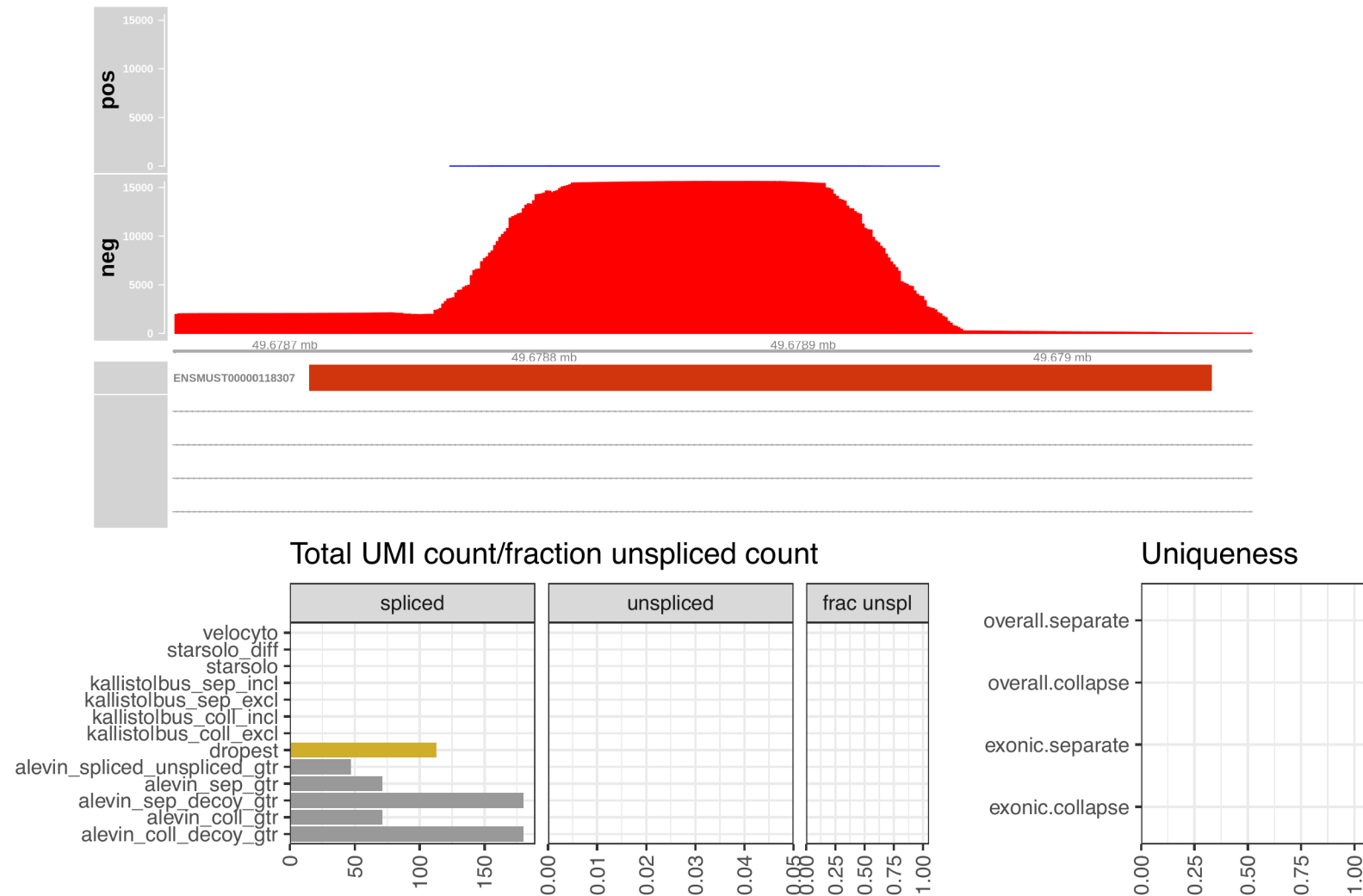

ENSMUSG00000054434 (Tmem120b)

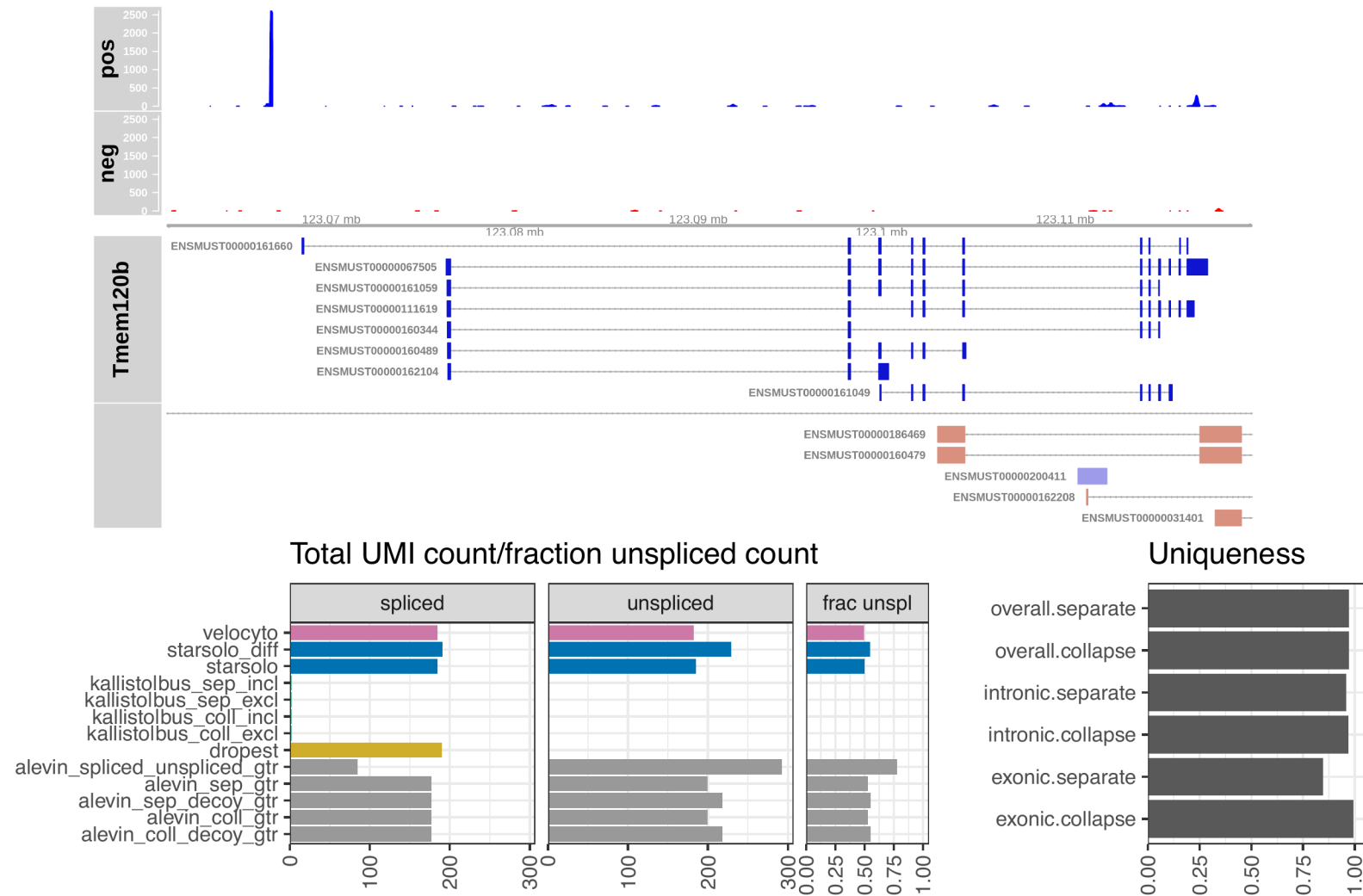

Supplement: S8 Fig — The coverage tracks show the number of reads overlapping each base position, on the positive (blue) or negative (red) strand. The isoforms of the main gene in focus of each panel are similarly shown in blue (positive strand) or red (negative strand). Any overlapping features from other genes are shown in the bottom annotation track, colored in muted blue or red, depending on the annotated strand. The bottom panels show the total exonic and intronic UMI count assigned to the displayed gene by the different quantification methods, as well as the fraction of unspliced counts, and the fraction of unique k-mers in the gene overall, as well as in the exons and introns. (PDF) [file pcbi.1008585.s008.pdf]

### Using genes selected by all methods

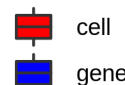

Supplement: S10 Fig — (PDF) [file pcbi.1008585.s010.pdf]

# Pancreas, fit likelihood vs average total abundance

For genes selected by all methods

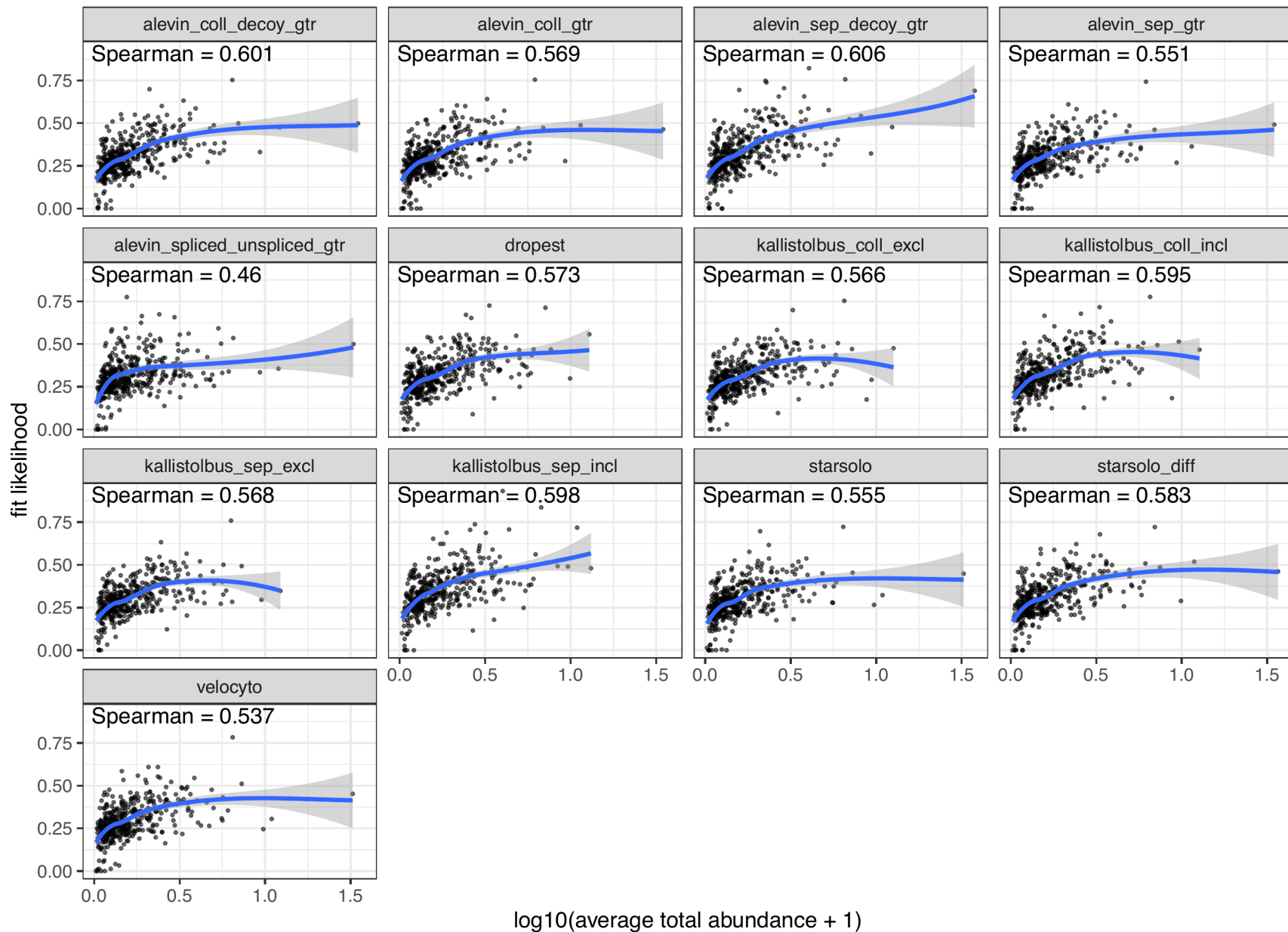

Supplement: S12 Fig — (PDF) [file pcbi.1008585.s012.pdf]

# Dentate gyrus, fit likelihood vs average total abundance

For genes selected by all methods

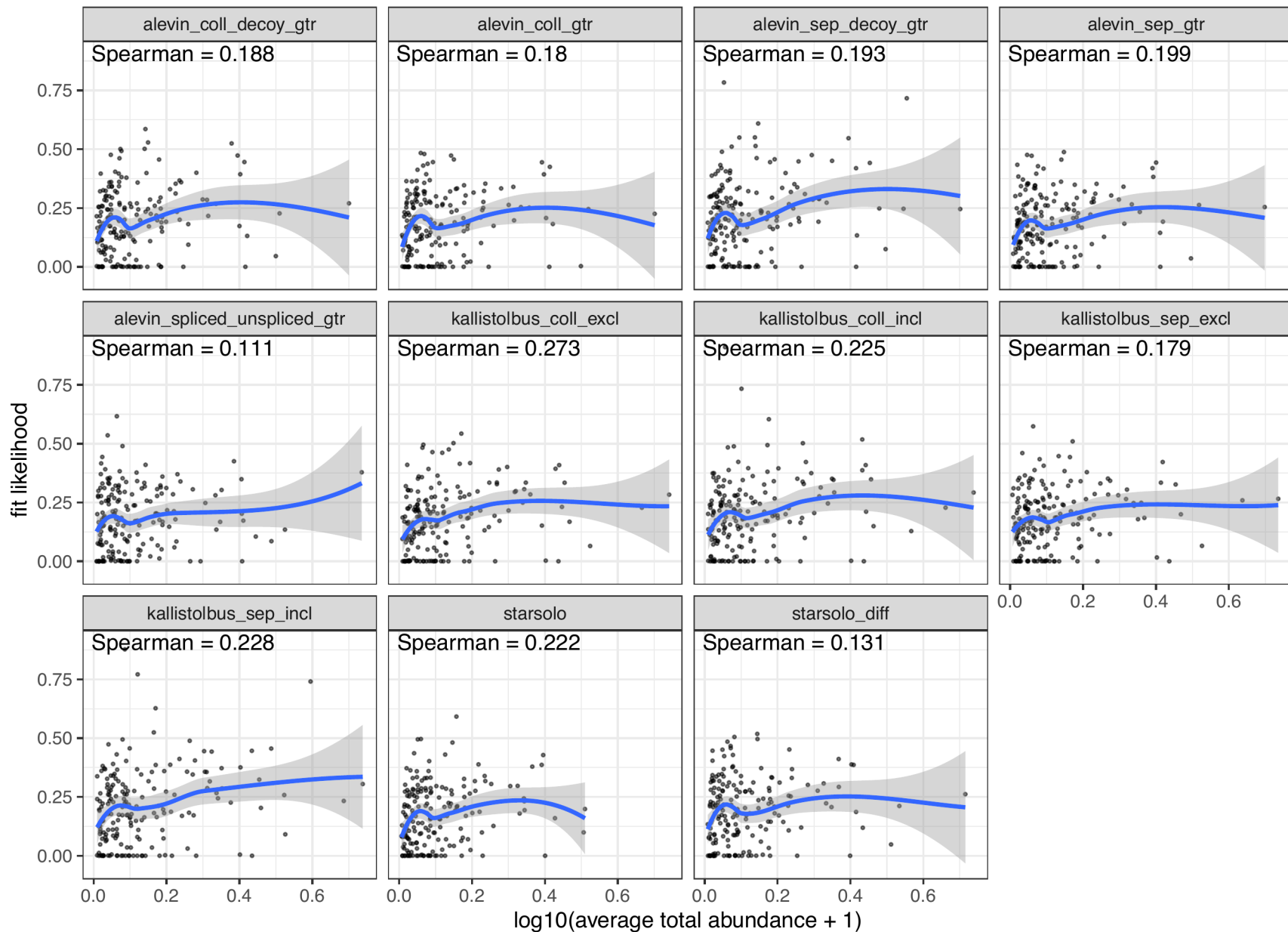

Supplement: S13 Fig — (PDF) [file pcbi.1008585.s013.pdf]

# Pancreas, length of embedded velocity vector

Embedded velocity length

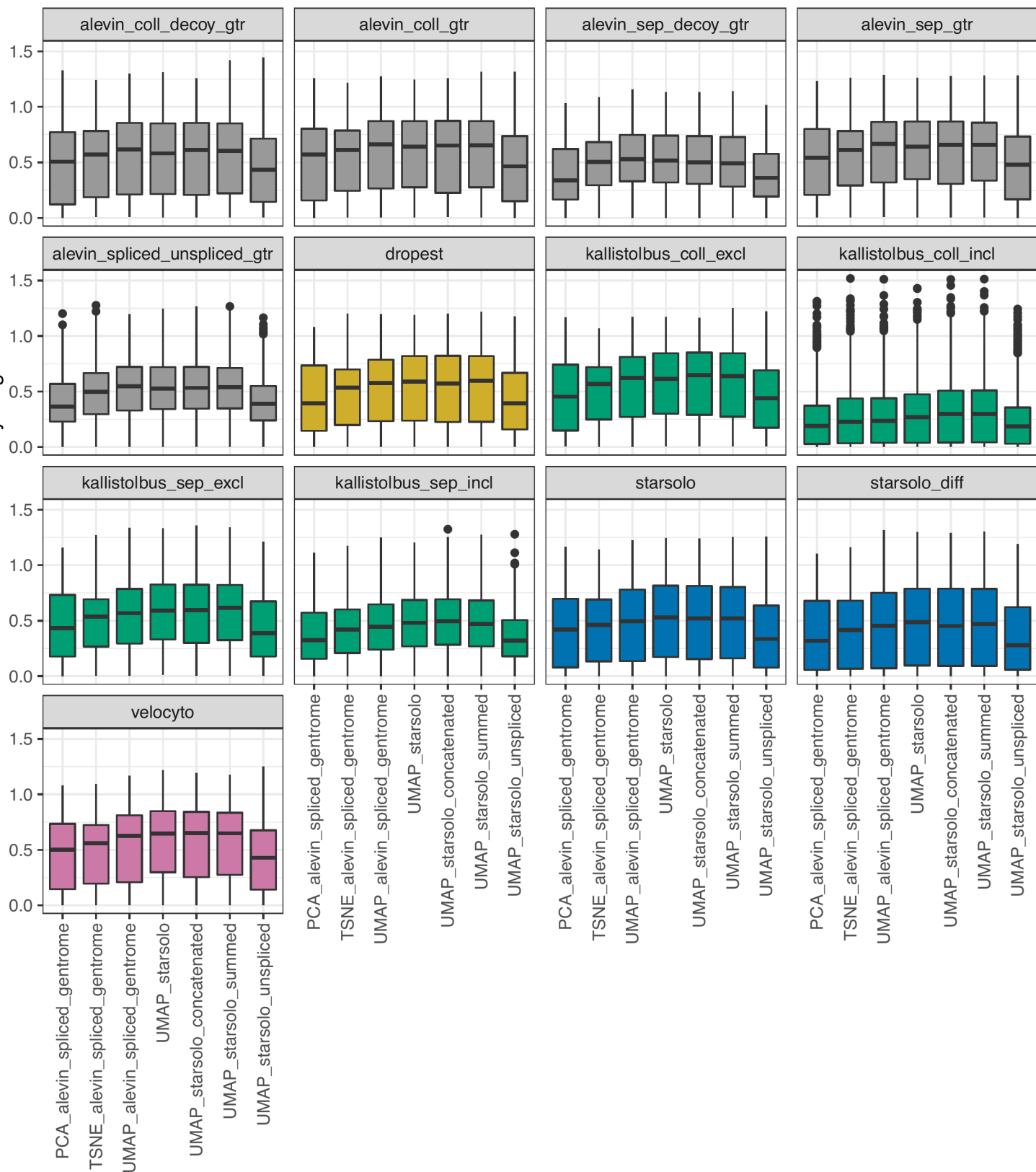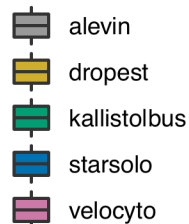

Supplement: S14 Fig — Since the displacement vectors in the low-dimensional representation are normalized before a weighted average is taken to determine the velocity embedding for a given cell, the length of the velocity embedding indicates to what extent the cells to which a cell i has a high transition probability are all located in the same direction from cell i in the low-dimensional representation. Consequently, the length of the embedded velocity vector provides one way of measuring how interpretable the low-dimensional representation is, from a velocity point of view. While overall only small differences are seen between dimension reductions, UMAP generally showed a small improvement in interpretability compared to PCA and TSNE, and using only the unspliced counts as the basis for the embedding was typically less informative. (PDF) [file pcbi.1008585.s014.pdf]

# Pancreas, average dot product with velocities of neighboring cells

Average dot product

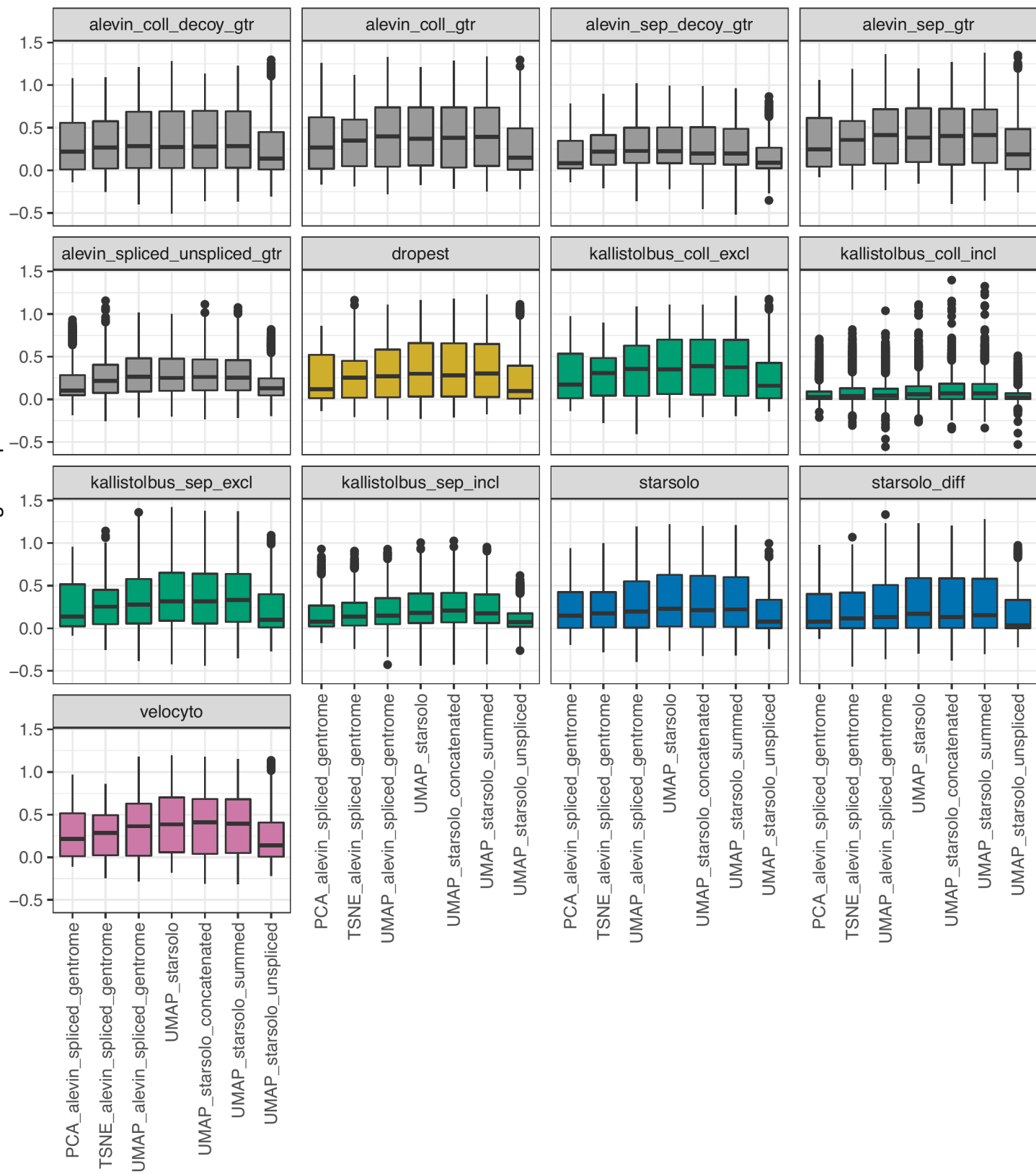

Supplement: S15 Fig — A high similarity suggests that velocity streamlines in the reduced dimension representation are more easily interpretable, since they are designed to summarize the dynamics across neighbouring cells. (PDF) [file pcbi.1008585.s015.pdf]
