# Supplementary material for: Preprocessing choices affect RNA velocity results for droplet scRNA-seq data
Source: PLoS Comput Biol. 2021 Jan 11;17(1):e1008585. doi: 10.1371/journal.pcbi.1008585 (PMC7822509; doi:10.1371/journal.pcbi.1008585)
Supplement: S3 Fig — In each case, the methods are compared to the methodologically most similar among the methods discussed in the main text. alevin_sep_flankLXX_gtr, with XX set to either 20 or 40, corresponds to running alevin with introns defined using the ‘separate’ approach, and using a flank length equal to the read length minus (XX+1). The alevin_sep_gtr method corresponds to setting XX = 0. Further, alevin_sep_gtr_unstranded corresponds to running alevin in unstranded mode. The kb-python wrapper uses the same type of intron definition and capture approach as kallisto|bus_sep_excl, but fixes the flank length to 30bp, whereas for kallisto|bus_sep_excl, the read length minus 1 was used. Top row: scatter plot of the total spliced and unspliced count assigned to genes with the different methods. Bottom row, left: Spearman correlation between abundances and velocities for each pair of methods. Bottom row, right: A classical multidimensional scaling (MDS) plot based on the Euclidean distances among velocity values for the set of shared genes. The various modifications to the methods have an impact on the derived velocities; however, the modified methods still cluster close to the corresponding base method. (PDF) [file pcbi.1008585.s003.pdf]

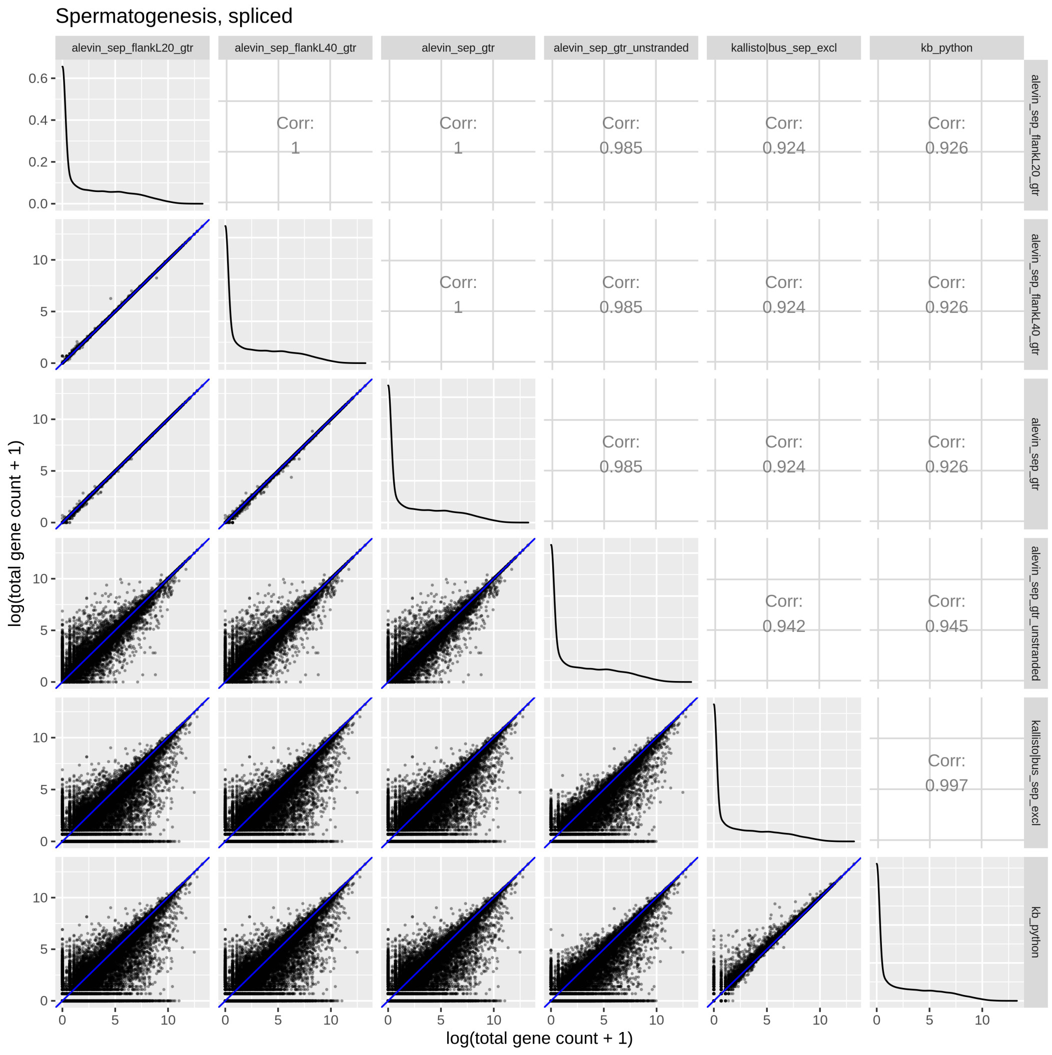
Spermatogenesis, spliced
alevin_sep_flankL20_gtr
alevin_sep_flankL40_gtr
alevin_sep_gtr
alevin_sep_gtr_unstranded
kallisto|bus_sep_excl
kb_python
Corr: 1
Corr: 1
Corr: 0.985
Corr: 0.924
Corr: 0.926
Corr: 1
Corr: 0.985
Corr: 0.924
Corr: 0.926
Corr: 0.985
Corr: 0.924
Corr: 0.926
Corr: 0.942
Corr: 0.945
Corr: 0.997
log(total gene count + 1)
log(total gene count + 1)

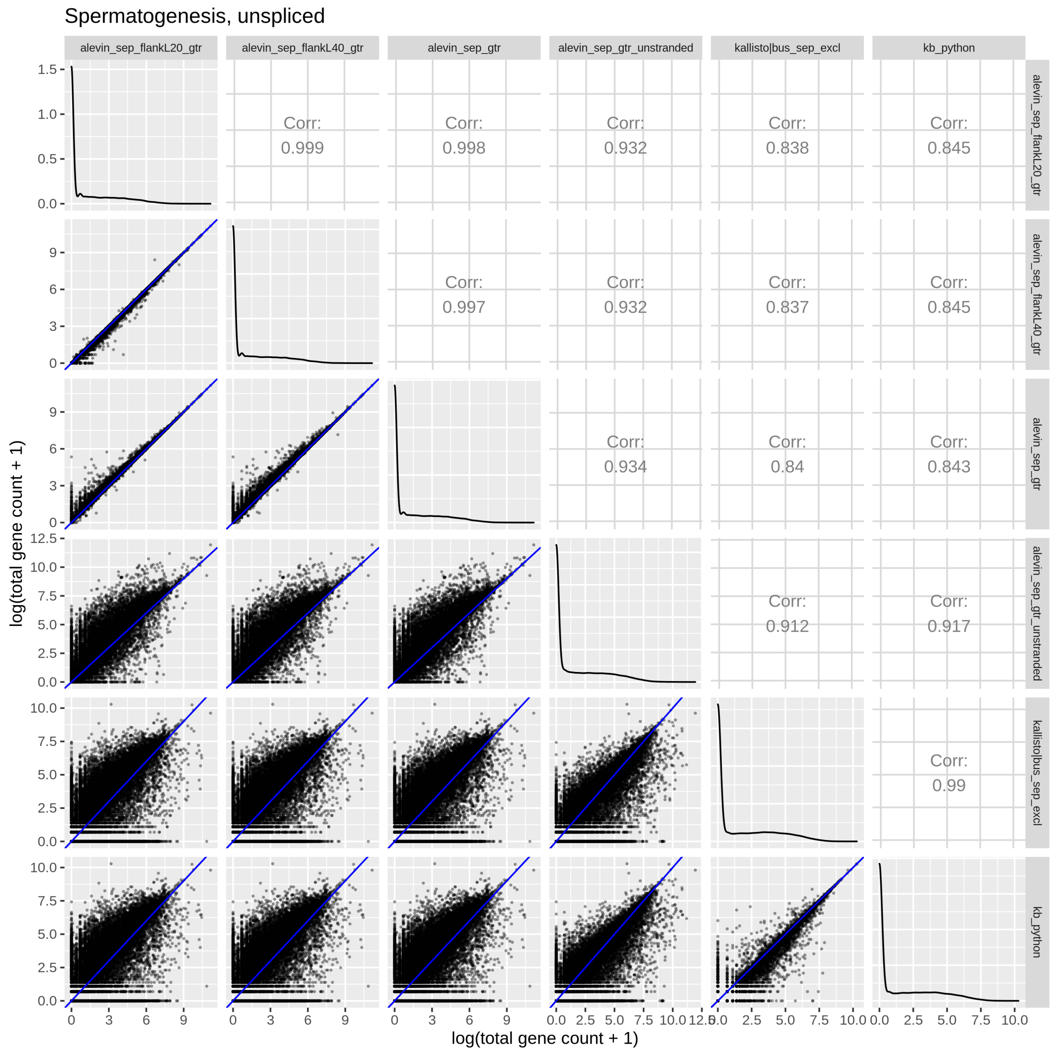
Spermatogenesis, unspliced
alevin_sep_flankL20_gtr
alevin_sep_flankL40_gtr
alevin_sep_gtr
alevin_sep_gtr_unstranded
kallisto|bus_sep_excl
kb_python
Corr: 0.999
Corr: 0.998
Corr: 0.932
Corr: 0.838
Corr: 0.845
Corr: 0.997
Corr: 0.932
Corr: 0.837
Corr: 0.845
Corr: 0.934
Corr: 0.84
Corr: 0.843
Corr: 0.912
Corr: 0.917
Corr: 0.99
log(total gene count + 1)
log(total gene count + 1)

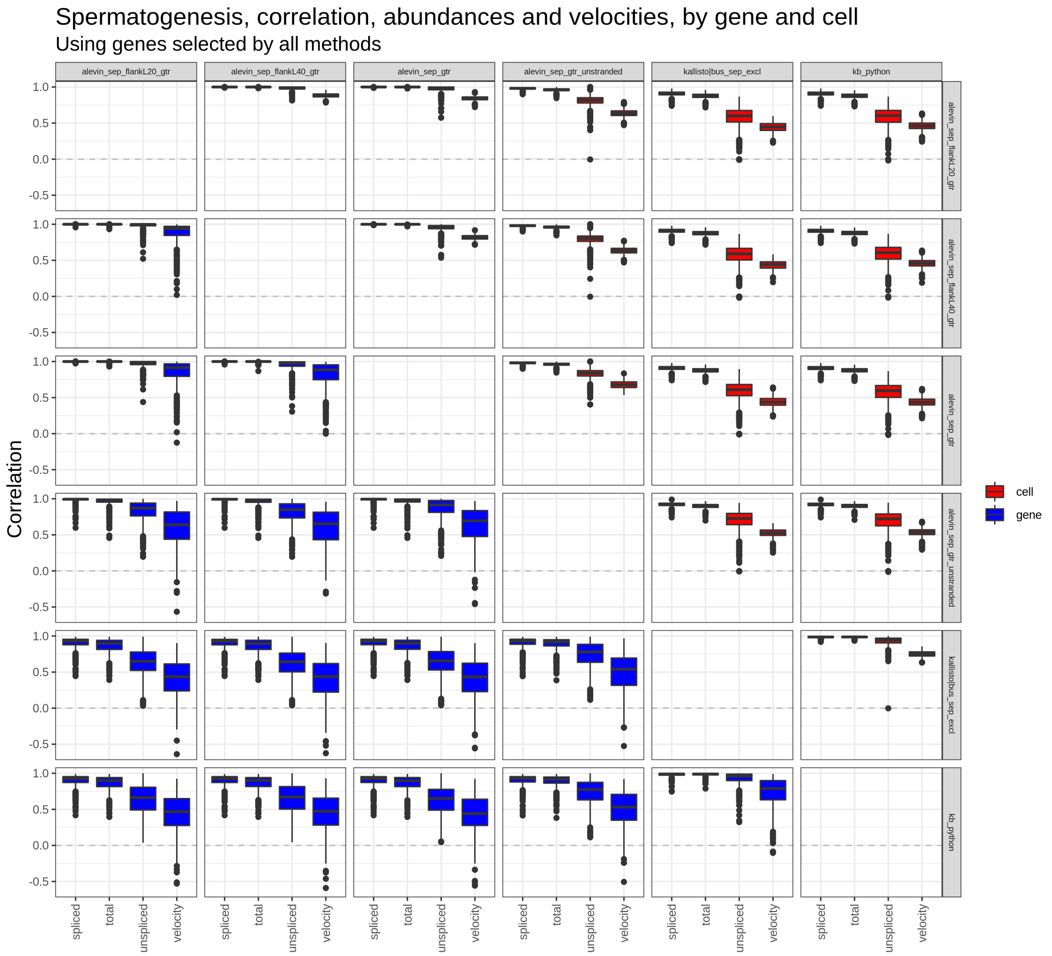
Spermatogenesis, correlation, abundances and velocities, by gene and cell
Using genes selected by all methods
alevin_sep_flankL20_gtr
alevin_sep_flankL40_gtr
alevin_sep_gtr
alevin_sep_gtr_unstranded
kallisto|bus_sep_excl
kb_python
Correlation
cell
gene
spliced
total
unspliced
velocity

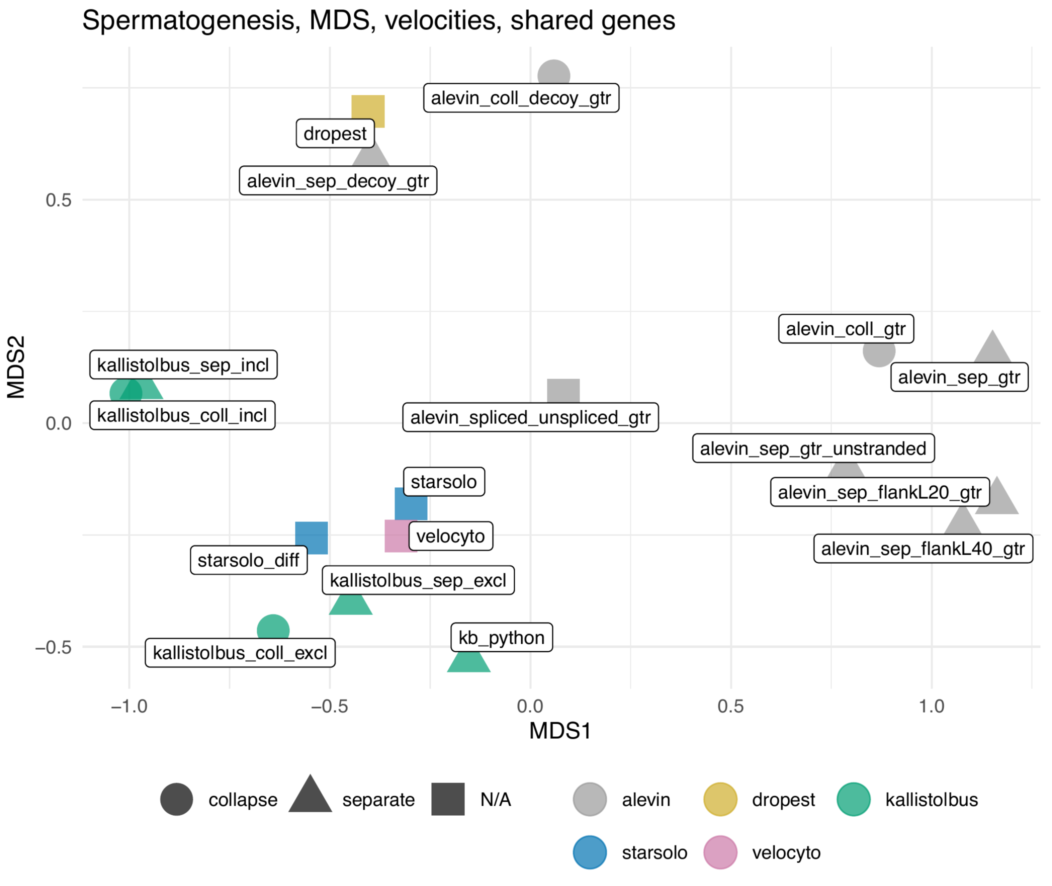
Spermatogenesis, MDS, velocities, shared genes
alevin_coll_decoy_gtr
dropest
alevin_sep_decoy_gtr
alevin_coll_gtr
alevin_sep_gtr
kallistolbus_sep_incl
kallistolbus_coll_incl
alevin_spliced_unspliced_gtr
alevin_sep_gtr_unstranded
alevin_sep_flankL20_gtr
alevin_sep_flankL40_gtr
starsolo
velocyto
starsolo_diff
kallistolbus_sep_excl
kallistolbus_coll_excl
kb_python
MDS1
MDS2
collapse
separate
N/A
alevin
dropest
kallistolbus
starsolo
velocyto
