# Supplementary material for: Preprocessing choices affect RNA velocity results for droplet scRNA-seq data
Source: PLoS Comput Biol. 2021 Jan 11;17(1):e1008585. doi: 10.1371/journal.pcbi.1008585 (PMC7822509; doi:10.1371/journal.pcbi.1008585)
Supplement: S9 Fig — Considered polyA/polyT stretches are at least 15 nt, with at most one mismatch per 15 nt in introns of genes on the forward and reverse strands. Similarly to [9], we observe consistent coverage around discordant internal priming regions. (PDF) [file pcbi.1008585.s009.pdf]

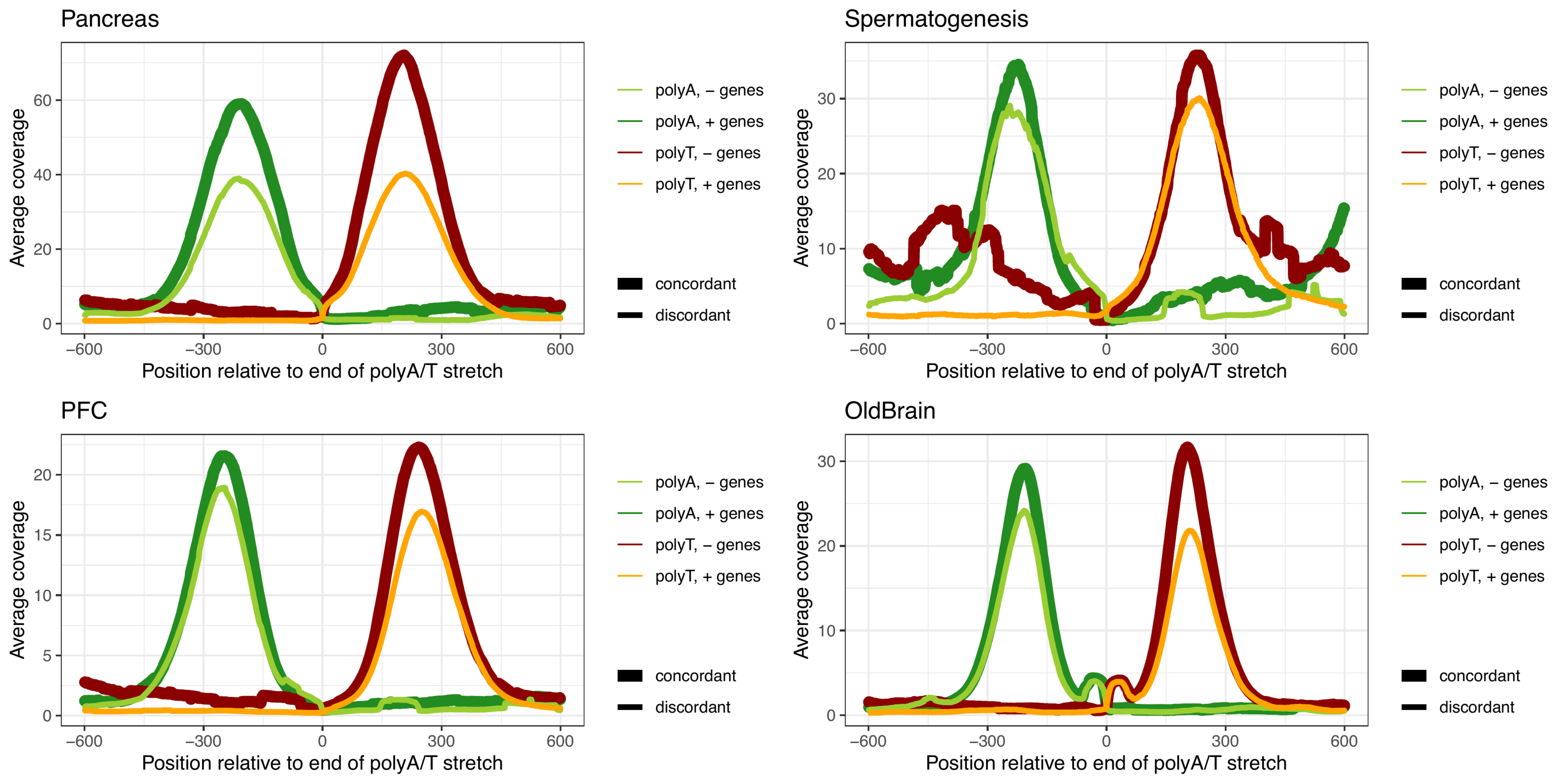

Pancreas
Spermatogenesis
PFC
OldBrain
Average coverage
Position relative to end of polyA/T stretch
polyA, – genes
polyA, + genes
polyT, – genes
polyT, + genes
concordant
discordant
