# Supplementary material for: Preprocessing choices affect RNA velocity results for droplet scRNA-seq data
Source: PLoS Comput Biol. 2021 Jan 11;17(1):e1008585. doi: 10.1371/journal.pcbi.1008585 (PMC7822509; doi:10.1371/journal.pcbi.1008585)
Supplement: S11 Fig — (PDF) [file pcbi.1008585.s011.pdf]

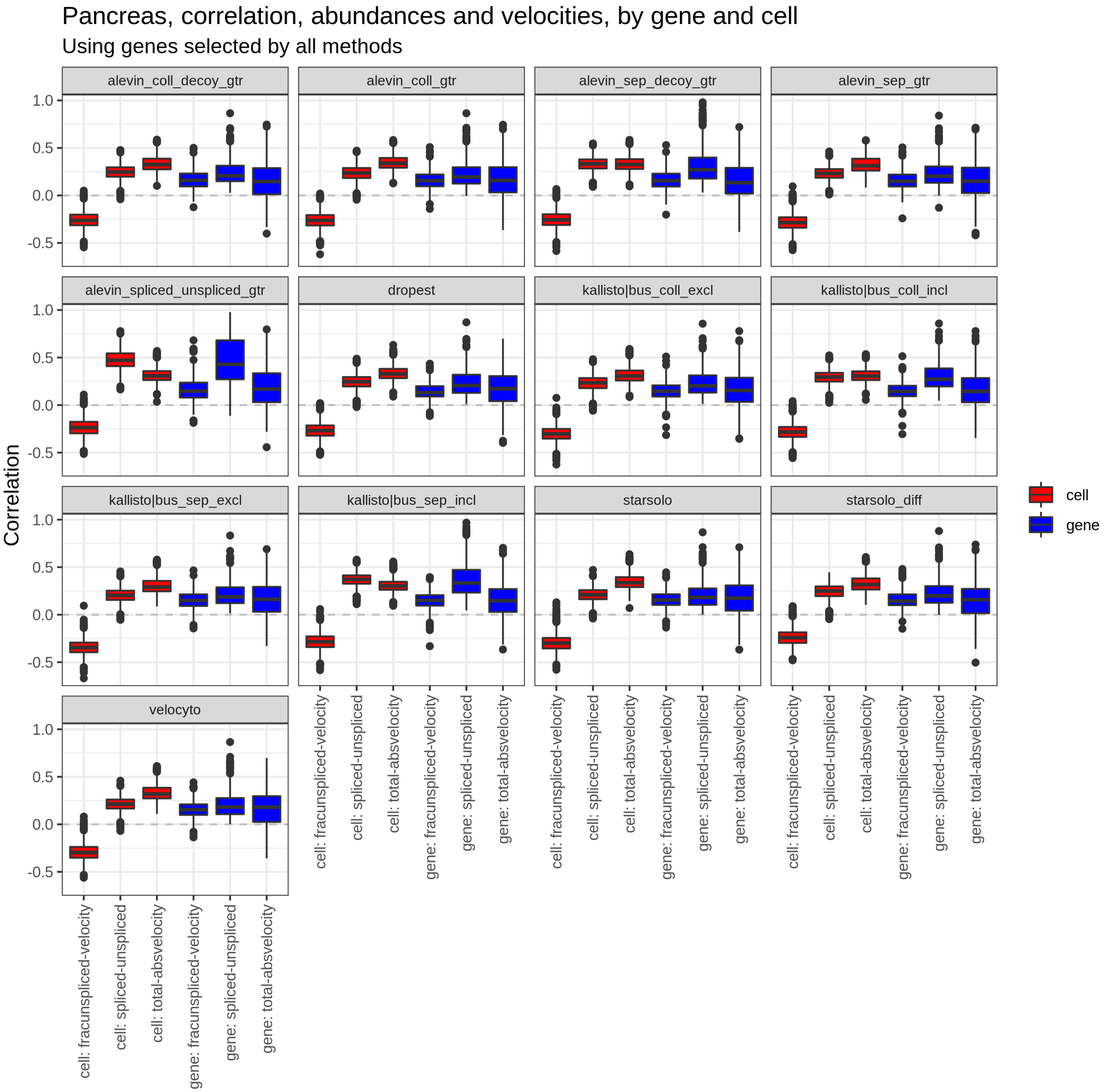

Pancreas, correlation, abundances and velocities, by gene and cell
Using genes selected by all methods
alevin_coll_decoy_gtr
alevin_coll_gtr
alevin_sep_decoy_gtr
alevin_sep_gtr
alevin_spliced_unspliced_gtr
dropest
kallisto|bus_coll_excl
kallisto|bus_coll_incl
kallisto|bus_sep_excl
kallisto|bus_sep_incl
starsolo
starsolo_diff
velocyto
Correlation
1.0
0.5
0.0
-0.5
cell
gene
cell: fracunspliced-velocity
cell: spliced-unspliced
cell: total-absvelocity
gene: fracunspliced-velocity
gene: spliced-unspliced
gene: total-absvelocity
