# Supplementary material for: Preprocessing choices affect RNA velocity results for droplet scRNA-seq data
Source: PLoS Comput Biol. 2021 Jan 11;17(1):e1008585. doi: 10.1371/journal.pcbi.1008585 (PMC7822509; doi:10.1371/journal.pcbi.1008585)
Supplement: S16 Fig — (PDF) [file pcbi.1008585.s016.pdf]

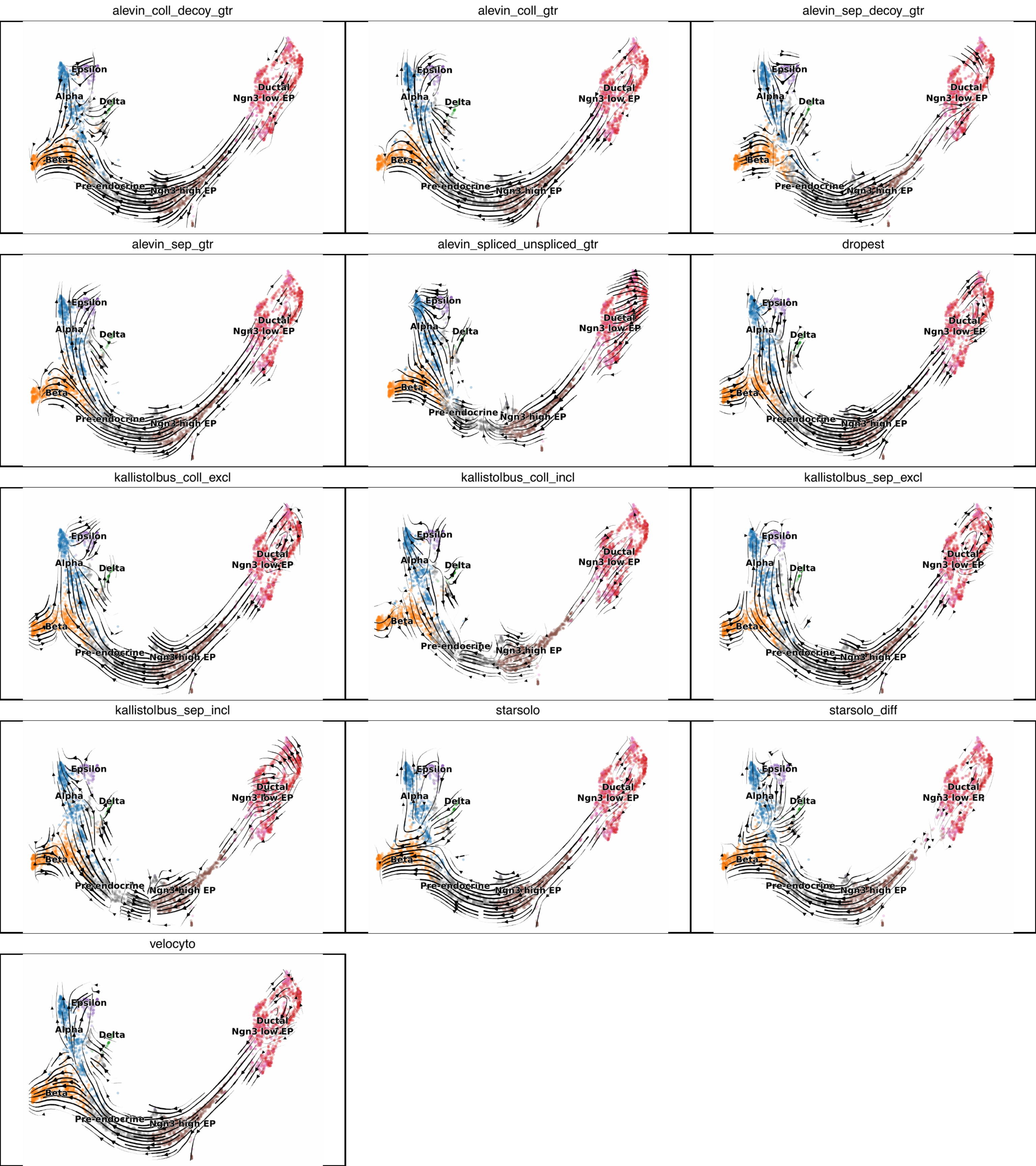
alevin_coll_decoy_gtr
alevin_coll_gtr
alevin_sep_decoy_gtr
alevin_sep_gtr
alevin_spliced_unspliced_gtr
dropest
kallistolbus_coll_excl
kallistolbus_coll_incl
kallistolbus_sep_excl
kallistolbus_sep_incl
starsolo
starsolo_diff
velocyto
Epsilon
Alpha
Delta
Beta
Pre-endocrine
Ngn3 high EP
Ductal
Ngn3 low EP
