# Supplementary material for: Preprocessing choices affect RNA velocity results for droplet scRNA-seq data
Source: PLoS Comput Biol. 2021 Jan 11;17(1):e1008585. doi: 10.1371/journal.pcbi.1008585 (PMC7822509; doi:10.1371/journal.pcbi.1008585)
Supplement: S18 Fig — (PDF) [file pcbi.1008585.s018.pdf]

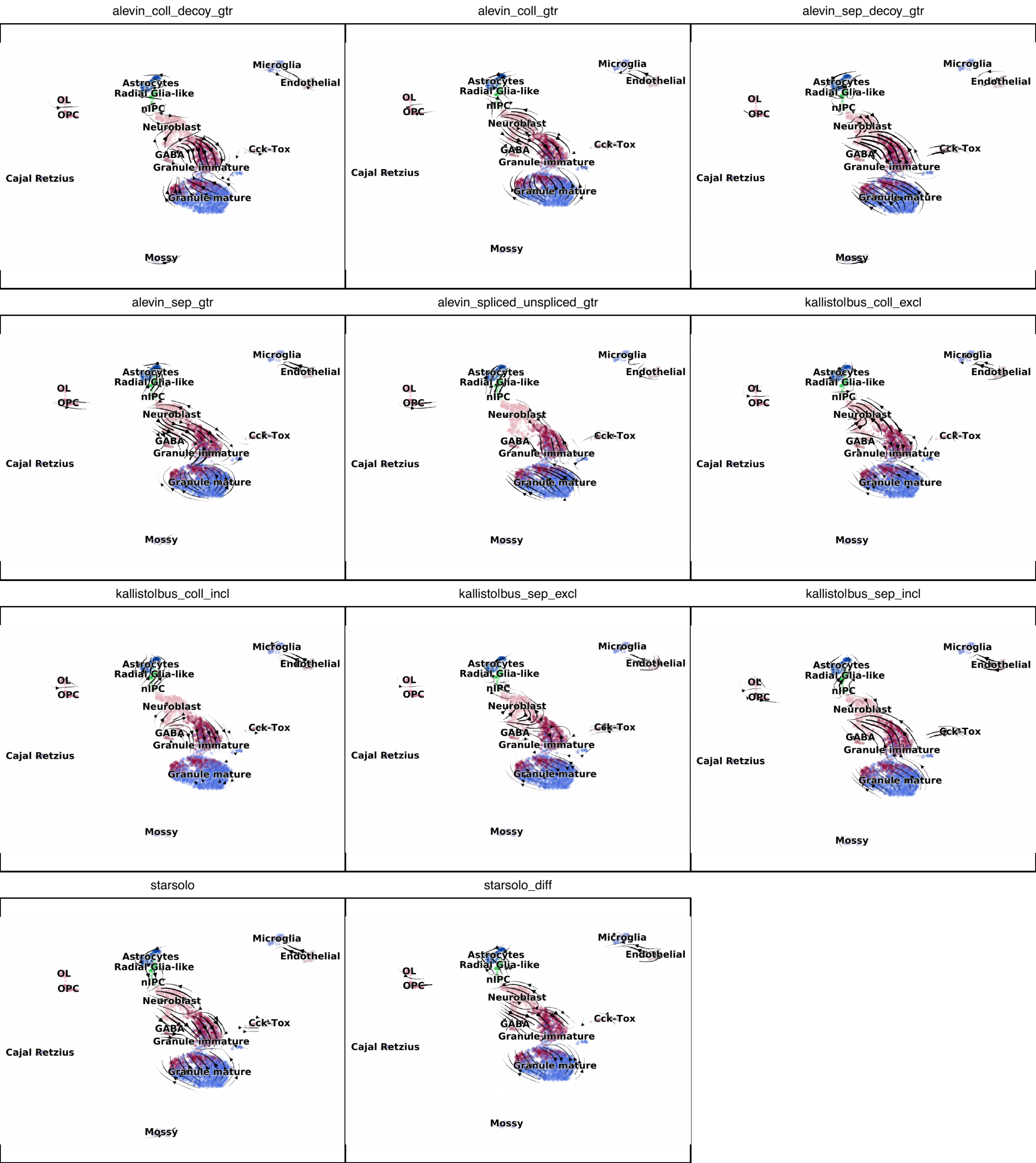
alevin_coll_decoy_gtr
Microglia
Endothelial
Astrocytes
Radial Glia-like
OL
OPC
nIPC
Neuroblast
Cck-Tox
GABA
Granule immature
Cajal Retzius
Granule mature
Mossy
alevin_coll_gtr
Microglia
Endothelial
Astrocytes
Radial Glia-like
OL
OPC
nIPC
Neuroblast
Cck-Tox
GABA
Granule immature
Cajal Retzius
Granule mature
Mossy
alevin_sep_decoy_gtr
Microglia
Endothelial
Astrocytes
Radial Glia-like
OL
OPC
nIPC
Neuroblast
Cck-Tox
GABA
Granule immature
Cajal Retzius
Granule mature
Mossy
alevin_sep_gtr
Microglia
Endothelial
Astrocytes
Radial Glia-like
OL
OPC
nIPC
Neuroblast
Cck-Tox
GABA
Granule immature
Cajal Retzius
Granule mature
Mossy
alevin_spliced_unspliced_gtr
Microglia
Endothelial
Astrocytes
Radial Glia-like
OL
OPC
nIPC
Neuroblast
Cck-Tox
GABA
Granule immature
Cajal Retzius
Granule mature
Mossy
kallistolbus_coll_excl
Microglia
Endothelial
Astrocytes
Radial Glia-like
OL
OPC
nIPC
Neuroblast
Cck-Tox
GABA
Granule immature
Cajal Retzius
Granule mature
Mossy
kallistolbus_coll_incl
Microglia
Endothelial
Astrocytes
Radial Glia-like
OL
OPC
nIPC
Neuroblast
Cck-Tox
GABA
Granule immature
Cajal Retzius
Granule mature
Mossy
kallistolbus_sep_excl
Microglia
Endothelial
Astrocytes
Radial Glia-like
OL
OPC
nIPC
Neuroblast
Cck-Tox
GABA
Granule immature
Cajal Retzius
Granule mature
Mossy
kallistolbus_sep_incl
Microglia
Endothelial
Astrocytes
Radial Glia-like
OL
OPC
nIPC
Neuroblast
Cck-Tox
GABA
Granule immature
Cajal Retzius
Granule mature
Mossy
starsolo
Microglia
Endothelial
Astrocytes
Radial Glia-like
OL
OPC
nIPC
Neuroblast
Cck-Tox
GABA
Granule immature
Cajal Retzius
Granule mature
Mossy
starsolo_diff
Microglia
Endothelial
Astrocytes
Radial Glia-like
OL
OPC
nIPC
Neuroblast
Cck-Tox
GABA
Granule immature
Cajal Retzius
Granule mature
Mossy
